# Quantification of T cell clonality in human T cell leukaemia virus type-1 carriers can detect the development of adult T cell leukaemia early

**DOI:** 10.1038/s41408-021-00458-8

**Published:** 2021-03-26

**Authors:** Sonia N. Wolf, Jana Haddow, Claire Greiller, Graham P. Taylor, Lucy B. M. Cook, Aileen G. Rowan

**Affiliations:** 1 Section of Virology, Department of Infectious Disease, Imperial College London, London, UK; 2 National Centre for Human Retrovirology, Imperial College Healthcare NHS Trust, London, UK; 3 Department of Haematology, Imperial College Healthcare NHS Trust, London, UK; 4 Centre for Haematology, Department of Immunology and Inflammation, Imperial College London, London, UK

**Keywords:** Tumour virus infections, Translational research, Leukaemia, Lymphoma

## Abstract

Adult T cell leukaemia/lymphoma (ATL) arises from clonally expanded T cells that are infected with human T cell leukaemia virus type-1 (HTLV-1). Here, we show that ATL can be detected early in HTLV-1-carriers through quantification of T-cell receptor (TCR)Vβ subunit diversity on T-cells infected with HTLV-1 (CD3+ CCR4+ CD26− T-cells) using an ‘oligoclonality index’ (OCI-flow). We established a reference range for OCI-flow by analysing peripheral blood mononuclear cells (PBMCs) from HTLV-1-carriers who had not developed ATL in a median of 10.5 years follow up (*n* = 38) and patients with ATL (*n* = 30). In the third cohort of HTLV-1-carriers with no history or clinical evidence of ATL (*n* = 106), 19% of high proviral load (PVL, ≥4 copies of HTLV-1/100 PBMCs) carriers had an OCI-flow in the ATL range, >0.770. Carriers with an OCI-flow >0.770 (*n* = 14) had higher lymphocyte counts and PVLs and were more likely to have a family history of ATL than carriers with OCI-flow ≤0.770. ATL subsequently developed in two of these 14 carriers but no carriers with OCI-flow ≤0.770 (*p* = 0.03, cumulative follow-up 129 person-years). This method can be used to identify a subset of high-PVL HTLV-1-carriers at increased risk of developing ATL who may benefit from intervention therapy, prior to the detection of disease.

## Background

There is an urgent clinical need for new approaches to improve the dismal prognosis of adult T cell leukaemia/lymphoma (ATL). The development of novel, well-tolerated therapeutics which can target both pre-malignant and malignant cells has raised the possibility of preventing ATL by treating those who are most at risk of transformation in the premalignant stage. These individuals can be identified through serological testing for human T cell leukaemia virus type 1 (HTLV-1), who have a 5% lifetime risk of developing ATL^1^. ATL occurs almost exclusively in carriers who have a high PVL, and thus have a ~20% lifetime risk of ATL^2^. Transformation generally occurs after many years of chronic infection, at a median age of ~50 in African-Caribbean and African–American patients^3,4^. Perinatal exposure to HTLV-1, family history of ATL, smoking, and high proviral load (PVL, ≥4 copies of HTLV-1 per 100 PBMCs) are well-established risk factors for transformation. We hypothesised that a novel diagnostic tool that identifies the premalignant stage of ATL in high PVL carriers could further risk stratify this group, by detecting the presence of premalignant cells prior to the onset of symptoms, thus enabling prediction of transformation and justifying therapeutic intervention to prevent disease development.

We and others have shown that malignant cells in ATL are derived from clonally expanded T cells which carry at least one copy of the HTLV-1 provirus integrated into the cellular genome^5,6^. Between 10^4^ and 10^5^ distinct infected T cell clones circulate in asymptomatic HTLV-1 carriers^7^. In contrast, at the time of ATL diagnosis, a single infected T cell clone dominates in 90% of cases^6^. Epigenetic and genetic changes confer enhanced fitness on the malignant ATL clone^8–10^, and drive an increase in the frequency of the transformed T cell clone, skewing the clone frequency distribution of HTLV-1-infected cells in the blood towards an oligoclonal or monoclonal distribution. Changes in clonality are an early biomarker of transformation: 42% of HTLV-1 carriers with ‘monoclonal’ populations of HTLV-1-infected cells in their peripheral blood (detected by low-resolution, semi-quantitative Southern blotting) developed ATL in a 20 year observation period (48 ATL cases/1000 carrier-years)^11^. To sensitively assess the degree of oligoclonality in the HTLV-1-infected T-cell population, linker-mediated polymerase chain reaction (LMPCR) and high-throughput sequencing (HTS) have been used to map and quantify HTLV-1 integration sites with high precision and depth^6,12–14^. This can be used to generate an oligoclonality index (OCI)^12^, derived from the Gini index^15^. This method is however costly, time-consuming and consequently difficult to translate into large-scale clinical application.

Surface molecules can be used to identify circulating HTLV-1-infected cells: they express C-C chemokine receptor type 4 (CCR4)^16^, cellular adhesion molecule 1 (CADM1)^17,18^ and are negative for CD26^19^. CD3+ CD4+ CCR4+ CD26− cells carry the bulk of the HTLV-1 proviral reservoir^20^. Downregulation of CD7 expression by CADM1+ cells appears to correlate with clonal expansion^21^ and individuals in whom 25–50% of CD4+ T cells are CADM1+ CD7dim/negative are reported to have a >50% chance of progressing from asymptomatic carrier to ATL in approximately 3 years^22^. However, this measure is a proxy and does not quantify clonality.

We have previously shown that the oligoclonality of HTLV-1 infected cells can be estimated through flow cytometric analysis of T cell receptor (TCR)Vβ subunit expression by CADM1+ T cells^23^. Oligoclonality scores generated by flow cytometry correlate with oligoclonality as measured by the gold standard technique, HTLV-1 integration site mapping, and can differentiate HTLV-1 carriers from ATL patients^23^. We applied this method to CD4+ CD3+ CCR4+ CD26− and CD8+ CD3+ CCR4+ CD26− PBMCs from ATL patients, and a cohort of HTLV-1 carriers who had not developed ATL during the 10 years from the tested sample. Antibodies specific for CCR4 and CD26 were used to identify HTLV-1-infected cells because antibodies to these cell surface markers are widely available and compatible with EuroFlow protocols^24^. This study has allowed us to establish a metric of oligoclonality within CD3+ CCR4+ CD26− cells which can identify individuals with oligoclonal expansions which may represent premalignant disease within HTLV-1 carriers and can be used to monitor the change in clonality in such patients over time.

## Methods

### Patient selection

Study participants were HTLV-1 carriers attending the National Centre for Human Retrovirology (Imperial College Healthcare NHS Trust, St. Mary’s Hospital, London). Written informed consent was obtained and research was conducted under the governance of the Communicable Diseases Research Group Tissue Bank, approved by the UK National Research Ethics Service (09/H0606/106, 15/SC/0089, 20/SC/0226). Viably preserved PBMCs were selected based on length of follow-up and outcome. A training cohort was assembled, in which the clinical outcome was known. This consisted of a ‘No-ATL’ group: HTLV-1 carriers, who had not developed ATL after a minimum of 7 years (median 10.5 years) follow-up since the baseline sample; and an ‘ATL’ group, consisting of patients with an ATL diagnosis. A ‘Screening’ cohort (*n* = 106) was also assembled, containing an unbiased cross-section of HTLV-1 carriers with any length of follow-up, who did not have ATL at the time of sample collection or previously. Further details of the cohort can be found in Supplementary Table 1.

### PBMC collection and storage

PBMC were isolated from whole blood by density-gradient centrifugation using histopaque-1077 (Sigma-Aldrich, St. Louis, MO, USA) from ethylenediaminetetraacetic acid-anticoagulated blood. Isolated PBMCs were washed twice in PBS, then cryopreserved in fetal calf serum (FCS) (Life Technologies, Paisley, UK) with 10% dimethylsulfoxide (Sigma-Aldrich) and stored at −150 °C until used.

### Proviral load assay

DNA was extracted from PBMCs as per the manufacturer’s instructions (DNeasy, Qiagen, Hilden Germany), and HTLV-1 proviral load quantified as previously described^2^.

### Flow cytometric staining

Cryopreserved PBMCs were thawed in PBS 10% FCS and washed once in PBS. Between 5 × 10^5^ and 2 × 10^6^ cells were placed in each of 8-wells of a 96-well plate and washed once in PBS. Cells were resuspended in Live/Dead stain (Zombie NIR^™^, Biolegend, San Diego, CA, USA) and incubated for 5 min in the dark. Cells were washed once with FACS buffer (7% v/v of normal goat serum in PBS) then incubated (20 min, room temperature) with antibodies (Supplementary Table 2) specific for CD3, CD4, CD8, CCR4, CD26, CD7 and 24 TCRVβ subunits (IO test Beta mark kit, Beckman Coulter, Brea, CA, USA). Cells were washed three times before fixing for 30 min with FoxP3 Fixation/Permeabilization buffer (eBioscience, San Diego, CA, USA). Cells were washed as before with permeabilization buffer, then incubated with anti-Ki-67 for 30 min at RT, then washed again with permeabilization buffer and stored in FACS buffer at 4 °C until analysis by flow cytometry. Between 500 and 5000 events were acquired in the live CD3+ CD4+ CCR4+ CD26− gate on a Becton Dickinson LSRFortessa.

### Analysis of oligoclonality

Data were analysed using Kaluza software (Beckman Coulter) using the gating strategy shown in Supplementary Fig. 1. The frequency of cells expressing each TCRVβ subunit was measured in four non-overlapping populations: (a: CD3+ CD4+ CCR4+ CD26− cells; b: all other CD3+ CD4+ T cells (‘Other CD4+’ T cells); c: CD3+ CD8+ CCR4+ CD26− T cells; d: all other CD3+ CD8+ T cells (‘Other CD8+’ T cells), and each was expressed as a percentage of total live CD3+ T cells. To estimate the frequency of T cells expressing Vβ subunits which were not recognised by antibodies in the panel (‘Off-panel’) within each of the four cell populations (a–d), the sum of frequencies of cells expressing all positively identified TCRVβ subunits was subtracted from the total frequency of the parent population within CD3+ T cells. Finally, the frequencies of 50 cellular populations were used to calculate the OCI of CD3+ CCR4+ CD26− cells (OCI-flow): 25 subsets of CD3+ CD4+ CCR4+ CD26− cells defined by TCRVβ expression (24 subsets expressing known TCRVβ subunits and one subset of cells expressing subunits which were off-panel), and the equivalent 25 subsets of CD3+ CD8+ CCR4+ CD26− cells. A type 1 correction for small sample size was applied as previously outlined in Turpin et al.^25^. The TCRVβ subunit “X” expressed by the most expanded population of HTLV-1-infected cells was identified by performing a within-individual comparison, to account for the inequality of distribution of TCRVβ subunit expression in uninfected T cells, as follows. First, the expected frequency of a TCRVβX within CD3+ CD4+ CCR4+ CD26− cells was calculated using the formula:

$$({\rm{TCRV}}\! \beta {\rm{X}}+^\prime{\rm{Other}}{\,\rm{CD}}4+^\prime/^\prime{\rm{Other}}{\rm{CD}}4+^\prime)\ast ({\rm{CD}}4+{\rm{CCR}}4+{\rm{CD}}26 \,\hbox{-})\ast100$$

where each phenotype denotes the observed frequency of cells expressing the respective markers as a percentage of live CD3+ cells. If the difference between the observed frequency of TCRVβX within CD3+ CD4+ CCR4+ CD26− cells and its expected frequency (calculated above) was >2% of total CD3+ cells (or >3% of total CD3+ cells in ‘off-panel’ TCRVβ subunits) that cell population was designated an ‘ATL-like’ clone in carriers or a ‘malignant clone’ in samples from ATL patients. These thresholds were chosen based on the frequency of premalignant ATL cells observed in genomic studies of the premalignant stage of ATL^10^. Reference ranges are shown in Supplementary Table 3.

### Analysis of CD7 and Ki-67 expression

CD3+ CD4+ cells were gated on the basis of CCR4, CD26 and TCRVβ subunit expression to identify three distinct populations: (1) the malignant or ATL-like clone (CCR4+ CD26− VβX+, if present); (2) cells bearing the rest of the proviral reservoir (CCR4+ CD26− VβX−) and (3) the remainder of CD3+ CD4+ T cells (‘Other CD4+’), which have a significantly lower proviral burden (Supplementary Fig. 2). CD7 and Ki-67 expression were quantified in each of the above populations. CD4+ cells were also analysed using a protocol adapted from the HAS-flow approach^21^, to measure the frequency of CD7^dim^CCR4+ CD26− (%D) and CD7− CCR4+ CD26− (%N) cells in total CD3+ CD4+ cells (Supplementary Fig. 2I).

### Statistical analysis

Statistical analysis was carried out using GraphPad Prism 8 version 8.1.2 for Windows, GraphPad Software, San Diego, CA, USA. Log-transformed data from the No-ATL cohort were used to calculate a reference range (the 95% prediction interval of the No ATL cohort, Supplementary Table 3). Transformation rates were calculated using incident cases divided by total follow-up time. As per convention, where subjects were lost to follow-up (did not attend the clinic for >2 years), 0.5 years was added to the denominator^26^. Positive or negative predictive values (PPV/NPV) were calculated as (true positives)/(true + false positives)*100. Univariate and multivariate logistic regression models were carried out on the binary outcome, OCI-flow ≤/> 0.770.

## Results

### Oligoclonality of HTLV-1-infected cells

Oligoclonality of HTLV-1-infected cells was assessed by quantifying TCRVβ subunit expression by CD4+ CD3+ CCR4+ CD26− and CD8+ CD3+ CCR4+ CD26− PBMCs in the training cohorts: (Table 1): the No-ATL cohort (*n* = 38 HTLV-1 carriers who did not transform during a median follow-up of 127 months, range: 84–225 months), and the ATL cohort (*n* = 30, all subtypes of ATL, Supplementary Table 1).

**Table 1:** Baseline demographic and clinical data from each group.

	ATL (*n* = 30)	No ATL (*n* = 38)	Screening (*n* = 106)
Ethnicity			
High risk^a^	30 (100%)	31 (82%)	96 (91%)
Low risk	0	7 (18%)	9 (8%)
Unknown	0	0	1 (1%)
Age			
Median (IQR)	60 (52–67)	55 (50–65)	55 (47–65)
Sex			
Male	10 (67%)	7 (18%)	29 (27%)
Female	20 (33%)	31 (82%)	77 (73%)
Diagnosis			
AC		17 (45%)	57 (54%)
HAM		18 (47%)	38 (36%)
Arthritis		0	2 (2%)
Neuropathy		1 (3%)	6 (6%)
Polymyositis		1 (3%)	0
Strongyloides-related disease		1 (3%)	2 (2%)
Uveitis		0	1 (1%)
Acute	9 (30%)		
Chronic	8 (27%)		
Lymphoma	11 (37%)		
Smouldering	2 (7%)		
Immunosuppressive therapy in past 6 weeks			
No		35 (92%)	89 (84%)
Yes		3 (8%)	17 (16%)
Family history			
No	29 (97%)	37 (97%)	93 (88%)
Possible^b^	1 (3%)	1 (3%)	5 (5%)
Yes	0	0	8 (8%)
Strongyloides status			
Negative	20 (67%)	22 (58%)	55 (52%)
Positive	4 (13%)	10 (26%)	8 (8%)
Unknown	6 (20%)	6 (16%)	43 (41%)
HIV status			
Negative	29 (97%)	38 (100%)	96 (91%)
Positive	1 (3%)	0	10 (9%)
PVL (% PBMCs)			
Median (IQR)	22.8 (4.9–45.9)	7.9 (4.2–13.0)	8.6 (2.9–15.5)
Lymphocytes (×10^9^/L)			
Median (IQR)	2.9 (1.2–6.3)	2.0 (1.6–2.3)	2.0 (1.6–2.5)
Missing data (*n*)	4	8	26

^a^High risk ethnicity is defined as patients of Caribbean, African, Middle Eastern, Asian, South American and Romanian descent, regardless of the place of birth.

^b^Possible family history indicates a family history of lymphoma/leukaemia where a diagnosis of ATL cannot be confirmed, usually due to diagnosis overseas.

**Fig. 1:**
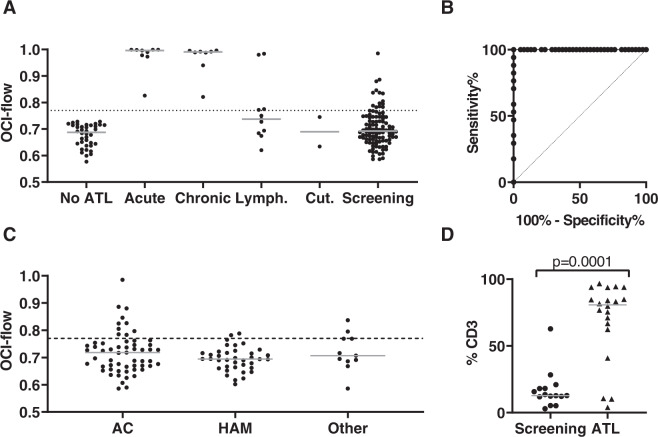
OCI-flow score and frequency of ATL-like clones. **a** OCI-flow scores of samples from the No-ATL (*n* = 38), ATL (*n* = 30) and screening cohorts (*n* = 106). Flow cytometric analysis was performed on PBMCs stained with a viability stain and antibodies specific for 24 TCRVβ subunits and T cell lineage markers. As there was no significant difference in the OCI-flow of treated vs untreated ATL patients in this cohort (Mann–Whitney test, *p* = 0.22) all ATL subjects have been included in this figure, regardless of whether they were on treatment or not. Lymph, lymphoma; Cut., cutaneous type ATL. Samples from patients with cutaneous subtype ATL were not included in the statistical analysis due to the low number of cases (*n* = 2). **b** Receiver-operating characteristic (ROC) curve of the ATL cohort and the no ATL cohort. Only ATL patients with blood disease (acute/chronic subtype ATL) were included. **c** OCI-flow of HTLV-1 carriers in the screening cohort by HTLV-1-related disease status. Subjects in the screening cohort were grouped into AC, HAM or HIV-coinfected (HIV) or ‘Other HTLV-associated diseases’ (Other) for statistical purposes (see Table 1). Statistical analysis: Kruskal–Wallis test. **d** Frequency of ATL-like and malignant clones within PBMC. The frequency of a given CCR4+ CD26− TCRVβX+ population was expressed as a percentage of total CD3+ cells in samples with OCI-flow >0.770. In the Screening cohort, *n* = 15 as three subjects have two populations of cells meeting our criteria or definition of a clone and two subjects had no clone meeting our criteria. In the ATL cohort, *n* = 20 as one subject had two populations meeting our definition of a clone. Statistical analysis: Mann Whitney, two-tailed. In all graphs, a grey line indicates the median.

Oligoclonality was quantified using the OCI-flow index, which ranges from 0 to 1: a value of 1 represents a perfectly monoclonal distribution (all cells express a single TCRVβ subunit) and 0 represents a perfectly polyclonal distribution (all TCRVβ subunits are present at equal frequencies). The median OCI-flow score of CD3+ CCR4+ CD26− PBMCs (see Methods) was 0.687 (range: 0.577–0.728) in the No-ATL cohort (Fig. 1A). These data were used to establish the limits of the OCI-flow reference range (0.590–0.770, Supplementary Table 3, “Materials and methods”). Testing of replicate samples showed that the assay was robust and reproducible with minimal variance between replicates (mean %CV = 0.97%, minimum 0.07%, maximum 2.6%, Supplementary Fig. 3).

OCI-flow was above the upper limit of the reference range (>0.770, median: acute, 0.996; chronic, 0.991) in all samples from patients with acute or chronic ATL, including those who started treatment before sample collection. The median OCI-flow in PBMCs from patients with lymphomatous and cutaneous subtypes was 0.737 and 0.690, respectively, indicating a lack of circulating lymphocyte involvement. Thirty-six per cent of patients with lymphoma subtype had an OCI-flow >0.770 (4/11 patients), indicating that malignant cells were probably circulating in the blood. Samples of lymphoma tissue were, however, not available to confirm whether lymphoma cells expressed the same TCRVβ subunit. Receiver operating characteristic (ROC) analysis of the No-ATL and ATL cohorts (acute/chronic subtypes only, *n* = 17) (Fig. 1B) showed 100% sensitivity and specificity using the cut-off OCI-flow = 0.770.

In the Screening cohort (*n* = 106, median follow-up 16 months, range: 0–135 months), there was no significant difference in the mean OCI-flow score between samples from asymptomatic carriers (AC), patients with HTLV-1-associated myelopathy (HAM), subjects co-infected with HIV, and subjects with other HTLV-associated diseases (Fig. 1C, *p* = 0.42, Kruskal–Wallis test).

### ATL-like HTLV-1 carriers: OCI-flow > 0.770 group

An OCI-flow > 0.770 was detected in PBMC from fourteen of the 106 subjects in the Screening cohort (13.2% of the total cohort, and 19.4% of high PVL HTLV-1 carriers (PVL ≥ 4%, *n* = 73)). Nine of these 14 subjects had a single population of TCRVβ-expressing cells which were expanded in CCR4+ CD26− cells relative to other T cells within that individual and also contributed >2% of CD3+ cells (here referred to as an ATL-like clone, see Materials and Methods for further details). Three subjects had two distinct ATL-like clones expressing different TCRVβ subunits. In subjects with an ATL-like clone, the median frequency of the clone was 13% of CD3+ cells (range: 3–63% of CD3+ cells, Fig. 1D). In the OCI-flow ≤ 0.770 group (*n* = 92), three subjects had populations of cells that met our criteria for an ATL-like clone. In the ATL cohort, 21 subjects had an OCI > 0.770, 19 of whom had populations of cells that met the same criteria for a malignant clone (17 patients with acute/chronic leukaemia and 2 with lymphoma), including one patient with 2 distinct clones. The median frequency of the malignant clone(s) in samples from the ATL cohort was 81% of CD3+ cells (range: 4–97%), which was significantly higher than the frequency of the ATL-like clones detected in the Screening cohort (Mann–Whitney test, *p* = 0.0001).

HTLV-1 carriers with an OCI-flow score > 0.770 were younger (median age 50 vs. 57 years, Mann–Whitney test *p* = 0.04, more likely to have a family history (confirmed/possible) of ATL (Fisher’s exact test, *p* = 0.01) and to have a higher lymphocyte count (Mann–Whitney test, *p* = 0.005) and proviral load *(p* = 0.0001) than carriers with OCI-flow ≤ 0.770 **(**Table 2). Lymphocyte counts were available for 11 individuals in this group, and two individuals had a count outside the normal range (>3.6 × 10^9^/L). There was no significant difference between the OCI-flow >0.770 and ≤0.770 groups in the frequency of subjects with parents from an endemic area (Fisher’s exact test, *p* = 0.6), female (*p* = 0.5); those with a diagnosis of HAM or another HTLV-associated disease (*p* = 0.25); those who were seropositive for *Strongyloides stercoralis (p* = 0.6); or those currently receiving immunosuppressive therapy for HAM (*p* = 1). Univariate logistic regression analysis showed that a high proviral load, a higher lymphocyte count and a family history of ATL were each independently associated with an OCI-flow >0.770, whereas greater age was inversely associated with OCI-flow > 0.770 (Supplementary Table 4A). A multivariate model tested in subjects with PVL ≥ 4% showed that age <50 and a lymphocyte count ≥2 × 10^9^/L were each independently significantly associated with OCI-flow > 0.770 (area under ROC 0.86, Supplementary Table 4B).

**Table 2:** Demographic and clinical data from the screening group, subdivided by OCI-flow score.

	OCI > 0.77	OCI ≤ 0.77	*p*
	*n* = 14	*n* = 92	
Ethnicity			
High risk	14 (15%)	82 (85%)	0.6
Low risk	0	9 (100%)	
Unknown	0	1 (100%)	
Age			
Median (IQR)	50 (40–57)	57 (48–66)	0.04
Sex			
Male	5 (17%)	24 (83%)	0.52
Female	9 (12%)	68 (88%)	
Diagnosis			
AC	10 (18%)	47 (82%)	0.25
HTLV-associated disease^a^	4 (8%)	45 (92%)	
Immunosuppressive therapy			
No	12 (13%)	77 (87%)	>0.99
Yes	2 (12%)	15 (88%)	
Family history			
No	9 (10%)	84 (90%)	0.01
Yes/possible	5 (38%)	8 (62%)	
Strongyloides status			
Negative	5 (9%)	50 (81%)	0.57
Positive	1 (13%)	7 (88%)	
Missing data (n)	8	35	
HIV status			
Negative	12 (13%)	84 (88%)	0.62
Positive	2 (20%)	8 (80%)	
PVL (% PBMCs)			
Median (IQR)	17.0 (13.9–21.5)	6.3 (2.2–12.9)	0.0001
Lymphocytes (×10^9^/L)			
Median (IQR)	2.5 (2.3–3.3)	1.9 (1.6–2.5)	0.005
Missing data (n)	3	23	

Statistical analysis was performed using Fisher’s exact test for categorical variables and Mann–Whitney for continuous variables.

^a^HTLV-associated disease includes in the OCI > 0.770 category: 2 HAM, 1 neuropathy, 1 uveitis. In the OCI ≤ 0.770 group, HTLV-associated disease includes 36 HAM, 5 neuropathy, 2 arthritis, 1 strongyloides, 1 uveitis.

### Clinical outcomes

Cumulative follow-up of subjects in the screening group was 178 person-years (129 person-years in carriers with PVL ≥ 4%). During follow-up, two subjects developed ATL, 41 months (acute) and 69 months (acute) after the screening sample was taken. In the screening cohort sample, the OCI-flow scores of PBMC from these patients were above the upper limit of the reference range: 0.985 and 0.846, respectively. One additional asymptomatic carrier with an OCI-flow of 0.880 who was considered high risk due to high HTLV-1 PVL, CD4 count and a family history of ATL was treated for 1 year with zidovudine and interferon-alpha although he did not meet ATL diagnostic criteria (normal lymphocyte count, serum lactate dehydrogenase and calcium). He remains well three years later, with a substantial reduction in OCI-flow (to 0.753) and a reduction in the frequency of the ATL-like clone from 18.1 to 2.6% of CD3+ cells.

Amongst subjects with PVL ≥ 4% (*n* = 72), those with OCI-flow > 0.770 were significantly more likely to transform to ATL (Fisher’s exact test, *p* = 0.03; positive likelihood ratio 6.3, 95% CI: 3.7–10.8) than subjects with OCI-flow ≤ 0.770 (despite the exclusion of the subject who underwent treatment intervention described above). The transformation rate among all carriers in our Screening cohort was 10.48 per 1000 person-years (95% CI: 2.6–41.6), and among carriers with a PVL ≥ 4% was 14.7 per 1000 person-years (95% CI: 3.7–58.3). Amongst carriers with an OCI-flow score > 0.770, the transformation rate was 72.5 per 1000 person-years (95% CI: 19.1–275.6).

### The likelihood of a high OCI-flow score increases with PVL

**Fig. 2:**
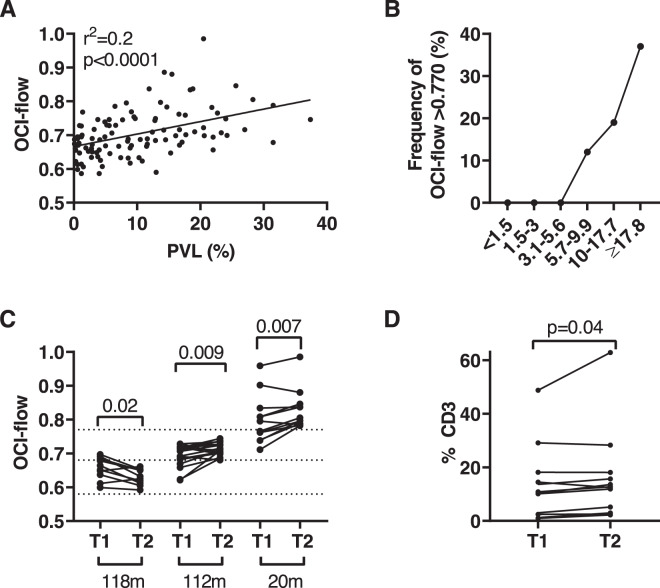
Relationship of proviral load with OCI-flow. **a** Proviral load and OCI-flow score of subjects in the screening cohort (*n* = 106). A simple linear regression line is plotted. **b** Frequency of subjects with an OCI-flow > 0.770. PVL is binned in 0.25log_10_ intervals. The number of subjects in each proviral load bin was: <1.5% (*n* = 21), 1.5–3 (*n* = 8), 3.1–5.6 (*n* = 15), 5.7–9.9 (*n* = 17), 10–17.7 (*n* = 26) and ≥17.8 (*n* = 19). **c** Temporal changes in OCI-flow. Subjects who were analysed longitudinally were binned by the OCI-flow score of the most recent sample: <0.680 (*n* = 10), 0.680–0.770 (*n* = 18) and >0.770 (*n* = 12) and tested for differences in OCI-flow between timepoint 1 (earliest) and time point 2 (latest). Statistical analysis: Wilcoxon test, two-tailed. **d** The frequency of ATL-like clones over time (*n* = 12, median interval = 20 months) was analysed using the Wilcoxon test, two-tailed.

In the least-squares regression model, both OCI-flow and the frequency of subjects in the Screening cohort who had OCI-flow > 0.770 increased as proviral load increased above 4% (Fig. 2A). No subject with a PVL < 4% had an OCI-flow > 0.770. In subjects with a PVL ≥ 17.8% (*n* = 19, 18% of Screening cohort), 37% of subjects had an OCI-flow > 0.770 (Fig. 2B). The observed non-linear increase in the percentage of subjects with OCI-flow > 0.770 resembles the rise in the risk of HAM with increasing PVL^27^.

### Longitudinal analysis

The stability of OCI-flow over time was assessed by analysing samples from 40 subjects at >1 timepoint (Fig. 2C). OCI-flow significantly increased over time in samples from subjects who had an OCI-flow > 0.770 at T2 (*n* = 12, median interval 20 months, Wilcoxon test, *p* = 0.007) and subjects who had an OCI-flow 0.680–0.770 at T2 (*n* = 18, median interval 112 months, *p* = 0.009). In subjects with OCI-flow < 0.680 (*n* = 10), OCI-flow decreased over time (median interval 118 months, *p* = 0.02). The frequency of the ATL-like clone in subjects with an OCI-flow > 0.770 also significantly increased between timepoints 1 and 2 (Fig. 2D).

### CD7 expression

**Fig. 3:**
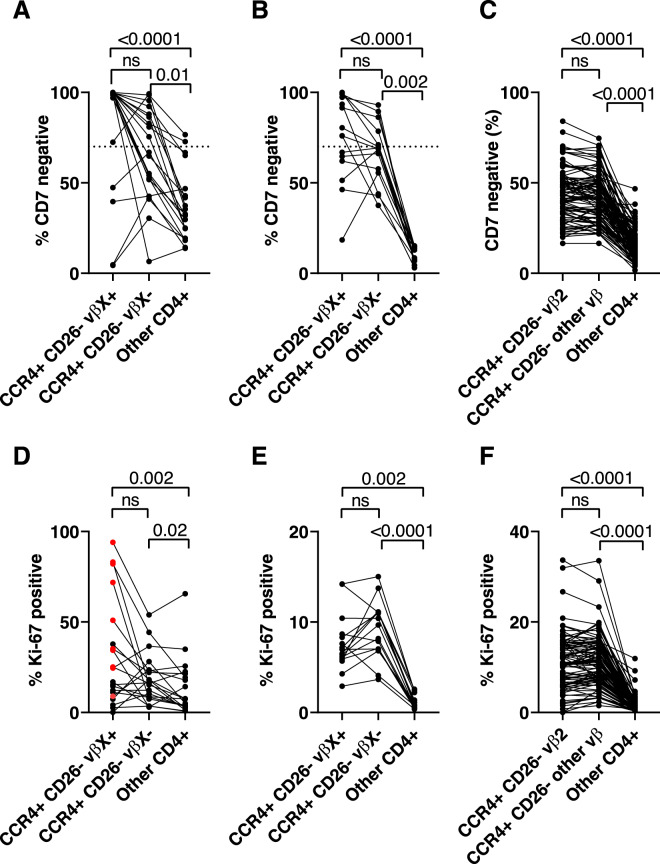
Within subject analysis of expression of CD7 and Ki-67. CD4+ T cells were divided into three non-overlapping populations on the basis of TCRVβ, CCR4 and CD26 expression: (1) if present, ATL-like clones were identified by gating on TCRVβX+ CCR4+ CD26− cells, (2) other HTLV-1 infected cells were identified by gating on TCRVβX− CCR4+ CD26− cells, and (2) all remaining CD4+ T cells were designated “Other CD4+ T cells”. CD7 and Ki-67 expression were quantified in each population. CD7 expression in samples with OCI-flow >0.770 in the ATL group (**a** *n* = 20 as 1 subject has two clones), the screening cohort (**b** *n* = 15; as three subjects had two expanded clones and two subjects did not have populations of cells which met our criteria for an ‘ATL-like’ clone) and **c** Screening cohort subjects with OCI-flow ≤ 0.770 (*n* = 92). Ki-67 expression in samples with OCI-flow > 0.770 in the ATL group (**d**) and the Screening cohort (**e**) and samples with OCI-flow ≤ 0.770 from the screening cohort (**f**). Statistical analysis: Friedman test with Dunn’s multiple comparisons test.

CD7 expression is commonly downregulated in ATL; however, it is also strongly downregulated in HTLV-1-infected cells in healthy carriers^23^: in the No-ATL cohort, the reference range of CD7− cells was 23.5–79.4% of CD4+ CCR4+ CD26− cells (Supplementary Table 3). In all subjects, expression of CD7 was significantly lower on CCR4+ CD26− cells compared with other CD4+ T cells (Fig. 3). However, in subjects with ATL and OCI-flow > 0.770, the frequency of CD7 expression by the malignant clone(s) (CCR4+ CD26− VβX+ cells, *n* = 20), was not significantly different from CD7 expression in CCR4+ CD26− VβX− cells (Friedman test, *p* = 0.46, Fig. 3A). In subjects in the Screening cohort with OCI-flow > 0.770, there was also no significant difference in the frequency of CD7− cells between the ATL-like clone and CCR4+ CD26− VβX− cells (*p* = 0.60 Fig. 3B). In Screening cohort subjects with OCI-flow ≤ 0.770 (*n* = 92), there was no difference in the frequency of cells expressing CD7 between a control population of TCRVβ2-expressing CCR4+ CD26− cells and other CCR4+ CD26− cells (*p* = 0.81, Fig. 3C).

### CD7 downregulation as a predictor of high OCI

We asked whether CD7 downregulation on CD3+ CD4+ CCR4+ CD26− cells could be used as a surrogate biomarker for a high OCI-flow score. We quantified the frequencies of CD7^dim^ and CD7− CD3+ CD4+ CCR4+ CD26− cells in total CD4+ cells in samples from the No-ATL and ATL cohorts (Supplementary Fig. 4) and the Screening cohort (Supplementary Fig. 4B, C). All ATL samples had ≥25% CD7dim/negative infected cells in CD4+ cells, however, 50% of samples from the No-ATL cohort also scored ≥25%, We further tested whether CD7 expression could differentiate samples with an OCI-flow >0.770 from samples with an OCI-flow ≤ 0.770 within the Screening cohort. Twelve of 14 samples with OCI-flow > 0.770 had >25% CD7dim/negative infected cells in CD4+ cells, however, 40 of 92 samples with OCI-flow ≤ 0.770 also scored >25% (PPV = 23%, NPV = 96%, Supplementary Fig. 4C). In the Screening cohort, the frequency of CD7 dim/negative infected cells within CD4+ cells was positively correlated with proviral load (*R*^2^ = 0.5, *p* < 0.001, Supplementary Fig. 4D) and OCI-flow (*R*^2^ = 0.36, *p* < 0.0001) (Supplementary Fig. 4E). Thus, we concluded that although CD7 downregulation was observed in individuals with ATL-like clones, it could not differentiate them from individuals with a high proviral load. In the screening cohort the transformation rate among carriers with ≥25% CD7 dim/negative cells was 21.9 per 1000 person-years (95% CI: 5.6–86.2), closer to the rate observed for high PVL carriers than the rate observed for OCI flow > 0.770 carriers.

### Ki-67 expression

CD4+ T cells which carry the bulk of the HTLV-1 proviral reservoir express high levels of the proliferation marker Ki-67. In all subjects, Ki-67 expression was significantly higher in CCR4+ CD26− cells than in other CD4+ T cells, and in the No-ATL cohort the reference range of Ki-67 expression was 5.7–23.1% of CCR4+ CD26− cells. However, in subjects with ATL and OCI > 0.770, the frequency of Ki-67 expression was not significantly higher in the malignant clone(s) compared to CCR4+ CD26−VβX− cells (*n* = 20, median 20.7% vs. 16.2%, *p* > 0.99, Fig. 3D), with the exception of samples from subjects with aggressive ATL (acute/lymphomatous subtypes, *n* = 12), which had a median frequency of Ki-67 expression of 34.8% in the malignant clone(s). In the Screening cohort, in subjects with OCI-flow > 0.770, the frequency of Ki-67 expression was not significantly different in the ATL-like clone(s) compared to the CCR4+ CD26− TCRVβX− cells (*p* = 0.60, Fig. 3E). In subjects with OCI-flow ≤ 0.770 (*n* = 92), there was also no significant difference in Ki-67 expression between CCR4+ CD26− Vβ2+ cells and other CCR4+ CD26− cells (*p* = 0.36, Fig. 3F).

## Discussion

For both clinicians and HTLV-1 carriers, the probability of developing ATL is of frequent concern. While the often-quoted figure of 5% is useful epidemiologically, it offers little to individual patients. Quantification of proviral load can further refine this estimate of lifetime risk, which reaches 20% in high PVL patients, but a lack of further known risk factors contributes to diagnostic and prognostic uncertainty in clinicians. The data presented here suggest carriers at very high risk of ATL can be identified and potentially offered interventions whilst low risk carriers can be reassured, including those with high PVL.

This study shows that flow cytometry, which is quantitative, fast and more widely accessible than LMPCR/HTS, can be used to derive an oligoclonality score for HTLV-1-infected cells which serves as a clinically useful prognostic and diagnostic index. Patients with leukaemic ATL have high OCI-flow scores. Carriers who did not develop ATL during >7 years’ follow-up all had an OCI-flow less than 0.770, whereas those with an OCI-flow >0.770 were over seven-fold more likely to develop the disease as compared to all carriers, and five times more likely to develop ATL than carriers with a PVL > 4%. This threshold for OCI-flow is supported by clinical and demographic differences between subjects. There are few data in patients with HTLV-1 on the impact of immunosuppressive therapy on the development of ATL. The lack of association observed in this study between high OCI-flow and immunosuppressive therapy is reassuring. Our regression model supports the use of known ATL risk factors in predicting high OCI-flow. However, these criteria alone are inadequate, because patients aged >50 with a peripheral blood lymphocyte count <2 × 10^9^/L do develop ATL, albeit at a lower frequency; therefore, we recommend assessment of clonality by flow cytometry for all carriers with a PVL ≥ 4%.

Approximately, 36% of patients with lymphomatous subtypes of ATL had an OCI-flow of >0.770, which may be an underestimate due to treatment prior to sampling in certain cases. In diagnosing lymphoma in an HTLV-1 carrier, the presence of a high OCI-flow would support a diagnosis of ATL over peripheral T-cell lymphoma not otherwise specified, while a negative result would not exclude it.

The transformation rate among all carriers observed here is comparable to that reported in a previous study (5.4 per 1000 person-years^28^ vs. 10.5 per 1000 person-years in this study). In carriers with OCI-flow > 0.770, the transformation rate (72.5 per 1000 person-years) is somewhat higher than previously reported data in which clonality was measured in ‘monoclonal’ patients^11^: this difference is likely due to the greater sensitivity of the OCI-flow method. Though further research is clearly warranted, the higher rate of transformation observed here suggests patients with OCI-flow > 0.770 require more intensive investigation and follow-up than their peers, because our data demonstrate a gradual increase in OCI-flow and clonal frequency over time—even within 1–2 years—in such patients. This observation supports previous reports of the evolution of clonality of HTLV-1-infected cells over time in asymptomatic carriers^12^. On the basis of the slow rate of change reported in Gillet et al.^12^, and the fact that an OCI > 0.8 (measured by LM-PCR) is observed in individuals 1–10 years prior to diagnosis with ATL^10^, it is now our practise to quantify the OCI-flow of HTLV-1 carriers with PVL ≥ 4% annually, and further evaluate those with an OCI-flow > 0.770. This may include more frequent follow-up (we suggest every 4 months), advise the patient to pay close attention to symptoms (e.g. skin rashes), and early clinical intervention, when the disease may be most susceptible to therapy; ideally treatment within clinical trials. The addition of further mutational profiling to detect high-risk driver mutations, which may be present up to 10 years prior to transformation^10^ may be beneficial, although is not currently in routine practice.

OCI-flow appears to be more specific for identifying patients with ‘ATL-like’ clones than methods which predict ATL development by measuring CD7 downregulation^21^, since CD7 downregulation increases with PVL, regardless of oligoclonality. Although CD7 expression analysis may replace assays of PVL in assessing ATL risk, they should not replace a direct measure of clonality, because the correlation between this method and OCI-flow is weak. OCI-flow can also predict transformation even when the absolute abundance of clones is small, whereas older techniques such as Southern blotting only detect clones of high abundance. In conclusion, OCI-flow is a practical tool to identify HTLV-1 carriers at high risk of ATL. We recommend annual OCI-flow testing in all HTLV-1 carriers with PVL ≥ 4%.

## Supplementary information

Supplemental Table 1

Supplemental material
