# Supplementary material for: Quantification of T cell clonality in human T cell leukaemia virus type-1 carriers can detect the development of adult T cell leukaemia early
Source: Blood Cancer J. 2021 Mar 26;11(3):66. doi: 10.1038/s41408-021-00458-8 (PMC7997885; doi:10.1038/s41408-021-00458-8)
Supplement: Supplementary file 1 — Supplemental Table 1 [file 41408_2021_458_MOESM1_ESM.pdf]

Sample	Anonymised ID	Group	OCI	OCI	No. ATL-like clones detected	Freq. of most expanded clone (% of CD3+ cells)	PVL at time of sample	Lymphocyte count	Diagnosis	Immunotherapy or chemo in past 6/52	Sex	Age at time of sample	Strongyloides	Ethnicity	FHx ATL	Outcome	Years follow up	Adjusted years follow up
238	ATL_1	Training-ATL	<0.770	0.620	0	n/a	2.1	0.7	ATL Lymphoma, HIV Co-infected	Post CHOP x 6, PEPC	m	68.0	Negative	Black		n/a	n/a	n/a
60	ATL_2	Training-ATL	>0.770	0.978	1	66.8	51.4	Missing data	Acute ATL	Prednisolone	f	74.0	Positive	Black		n/a	n/a	n/a
47	ATL_3	Training-ATL	>0.770	0.989	1	83.2	21.7	3.7	Chronic ATL	Zidovudine/Interferon alpha	f	64.0	Negative	Black		n/a	n/a	n/a
24	ATL_4	Training-ATL	>0.770	0.999	1	93.9	37.6	22.8	Acute ATL	No Rx	f	76.0	Unknown	Black		n/a	n/a	n/a
65	ATL_5	Training-ATL	<0.770	0.634	0	n/a	1.7	Missing data	Cutaneous ATL	No Rx	f	56.0	Negative	Black		n/a	n/a	n/a
241	ATL_6	Training-ATL	>0.770	0.775	0	n/a	5.6	1.6	ATL Lymphoma	#2 chemo (gem/Cis x 1, Gem/Carb x 1, DXT, ZDV/IFN)	f	62.0	Unknown	Black		n/a	n/a	n/a
61	ATL_7	Training-ATL	>0.770	0.940	1	40.9	17.9	3.2	Chronic ATL	No Rx	f	58.0	Negative	Black		n/a	n/a	n/a
57	ATL_8	Training-ATL	>0.770	0.996	1	93.8	42.1	12.2	Chronic ATL	No Rx	m	51.0	Positive	Black		n/a	n/a	n/a
242	ATL_9	Training-ATL	<0.770	0.737	0	n/a	7.9	1.0	ATL Lymphoma	#6 GemCar	f	63.0	Unknown	Black		n/a	n/a	n/a
74	ATL_10	Training-ATL	>0.770	0.821	1	10.7	22.1	1.5	Chronic ATL	Multiple lines chemothrapy/Mogamulizumab	f	57.0	Negative	Black		n/a	n/a	n/a
58	ATL_11	Training-ATL	>0.770	0.973	2	71.1	181.0	42.2	Acute ATL	Zidovudine/Interferon alpha	f	71.0	Negative	Black		n/a	n/a	n/a
233	ATL_12	Training-ATL	<0.770	0.684	0	n/a	2.2	0.5	ATL Lymphoma	Post #4 CHOP	m	67.0	Negative	Black		n/a	n/a	n/a
59	ATL_13	Training-ATL	>0.770	0.996	1	84.6	37.2	0.8	Acute ATL	3 x CHOP, Zidovudine	f	61.0	Unknown	Black		n/a	n/a	n/a
51	ATL_14	Training-ATL	>0.770	0.990	1	62.4	54.5	3.1	Acute ATL	Mogamulizumab	m	61.0	Positive	Black	possible	n/a	n/a	n/a
234	ATL_15	Training-ATL	<0.770	0.727	0	n/a	10.9	1.3	ATL Lymphoma	3 x CHOP, Zidovudine then stopped 6/9/16	f	59.0	Unknown	Black		n/a	n/a	n/a
55	ATL_16	Training-ATL	<0.770	0.750	0	n/a	3.5	2.5**	ATL Lymphoma	No Rx	m	53.0	Negative	Black		n/a	n/a	n/a
52	ATL_17	Training-ATL	>0.770	0.772	0	n/a	4.7	1.2	ATL Lymphoma	No Rx	f	54.0	Negative	Black		n/a	n/a	n/a
53	ATL_18	Training-ATL	>0.770	0.999	1	96.7	56.1	6.3	Acute ATL	No Rx	m	39.0	Negative	Black		n/a	n/a	n/a
54	ATL_19	Training-ATL	<0.770	0.745	0	n/a	1.9	2.1	Cutaneous ATL	No Rx	f	85.0	Negative	Black		n/a	n/a	n/a
69	ATL_20	Training-ATL	>0.770	0.995	1	84.9	56.0	10.8	Chronic ATL	No Rx	m	59.0	Negative	Black		n/a	n/a	n/a
235	ATL_21	Training-ATL	<0.770	0.694	0	n/a	6.7	2.7***	ATL Lymphoma	Post #6 CHOP	f	49.0	Negative	Black		n/a	n/a	n/a
48	ATL_22	Training-ATL	>0.770	0.984	1	75.4	23.4	3.1	ATL Lymphoma	No Rx	f	68.0	Negative	Black		n/a	n/a	n/a
49	ATL_23	Training-ATL	>0.770	0.992	1	79.8	88.6	6.0	Chronic ATL	No Rx	f	37.0	Negative	Black		n/a	n/a	n/a
64	ATL_24	Training-ATL	>0.770	0.999	1	94.4	99.5	9.7	Acute ATL	Zidovudine/Interferon alpha	m	51.0	Negative	Black		n/a	n/a	n/a
236	ATL_25	Training-ATL	>0.770	0.980	1	81.7	39.7	3.0	ATL Lymphoma	CHOP x 4, ICE- refractory	f	35.0	Negative	Black		n/a	n/a	n/a
237	ATL_26	Training-ATL	<0.770	0.674	0	n/a	4.4	1.2	ATL Lymphoma	Post #2 CHOP	f	65.0	Positive	Black		n/a	n/a	n/a
50	ATL_27	Training-ATL	>0.770	0.979	1	93.9	46.1	Missing data	Acute ATL	No Rx	m	66.0	Negative	Middle Eastern		n/a	n/a	n/a
67	ATL_28	Training-ATL	>0.770	0.826	1	10.2	3.4	0.3	Acute ATL	Cycle 5 day 14 CHOP	f	46.0	Negative	Black		n/a	n/a	n/a
215	ATL_29	Training-ATL	>0.770	0.988	1	84.1	45.4	9.6	Chronic ATL	No Rx	f	35.0	Negative	Other		n/a	n/a	n/a
230	ATL_30	Training-ATL	>0.770	0.992	1	76.8	36.2	Missing data	Chronic ATL	Zidovudine only	m	69.0	Unknown	Middle Eastern		n/a	n/a	n/a
39	No_ATL_1	Training-No_ATL	<0.770	0.724	0	n/a	15.3	1.4	AC	No Rx	f	73.0	Negative	Black		n/a	n/a	n/a
32	No_ATL_2	Training-No_ATL	<0.770	0.697	0	n/a	9.0	1.8	AC	No Rx	f	67.0	Unknown	Middle Eastern		n/a	n/a	n/a
240	No_ATL_3	Training-No_ATL	<0.770	0.720	0	n/a	15.2	2.2	AC	No Rx	f	32.0	Negative	Black		n/a	n/a	n/a
25	No_ATL_4	Training-No_ATL	<0.770	0.664	0	n/a	0.9	2.0	AC	No Rx	f	69.0	Positive	Black		n/a	n/a	n/a
239	No_ATL_5	Training-No_ATL	<0.770	0.645	0	n/a	3.9	2.6	AC	No Rx	m	49.0	Negative	Black		n/a	n/a	n/a
41	No_ATL_6	Training-No_ATL	<0.770	0.707	0	n/a	4.7	1.6	AC	No Rx	f	54.0	Unknown	Black	possible	n/a	n/a	n/a
246	No_ATL_7	Training-No_ATL	<0.770	0.682	0	n/a	6.7	1.0	AC	No Rx	f	54.0	Unknown	Black		n/a	n/a	n/a
5	No_ATL_8	Training-No_ATL	<0.770	0.641	0	n/a	1.9	2.3	AC	No Rx	f	50.0	Positive	Black		n/a	n/a	n/a
3	No_ATL_9	Training-No_ATL	<0.770	0.616	0	n/a	0.1	Missing data	AC	No Rx	f	39.0	Negative	Black		n/a	n/a	n/a
6	No_ATL_10	Training-No_ATL	<0.770	0.577	0	n/a	3.0	1.5	AC	No Rx	f	38.0	Negative	Indian/Pakistani/Bangladeshi		n/a	n/a	n/a
40	No_ATL_11	Training-No_ATL	<0.770	0.605	0	n/a	1.9	1.6	AC	No Rx	f	44.0	Negative	Black		n/a	n/a	n/a
13	No_ATL_12	Training-No_ATL	<0.770	0.611	0	n/a	7.9	3.4	AC	No Rx	f	57.0	Negative	White		n/a	n/a	n/a
7	No_ATL_13	Training-No_ATL	<0.770	0.728	0	n/a	27.9	1.6	AC	No Rx	f	67.0	Negative	Black		n/a	n/a	n/a
28	No_ATL_14	Training-No_ATL	<0.770	0.716	0	n/a	7.2	Missing data	AC	No Rx	m	21.0	Negative	White		n/a	n/a	n/a
8	No_ATL_15	Training-No_ATL	<0.770	0.669	0	n/a	6.6	1.1	AC	No Rx	f	50.0	Positive	Black		n/a	n/a	n/a
164	No_ATL_16	Training-No_ATL	<0.770	0.658	0	n/a	2.5	2.3	AC	No Rx	f	62.0	Negative	White		n/a	n/a	n/a
166	No_ATL_17	Training-No_ATL	<0.770	0.637	0	n/a	1.9	1.4	AC	No Rx	f	41.0	Unknown	White		n/a	n/a	n/a
250	No_ATL_18	Training-No_ATL	<0.770	0.728	0	n/a	8.4	Missing data	N	No Rx	f	55.0	Negative	Black		n/a	n/a	n/a
251	No_ATL_19	Training-No_ATL	<0.770	0.715	0	n/a	12.4	2.2	P	No Rx	f	62.0	Negative	Black		n/a	n/a	n/a
232	No_ATL_20	Training-No_ATL	<0.770	0.714	0	n/a	12.9	Missing data	S	No Rx	f	59.0	Positive	Black		n/a	n/a	n/a
9	No_ATL_21	Training-No_ATL	<0.770	0.599	0	n/a	11.4	1.8	HAM	No Rx	f	63.0	Positive	Black		n/a	n/a	n/a
243	No_ATL_22	Training-No_ATL	<0.770	0.716	0	n/a	13.0	Missing data	HAM	No Rx	f	69.0	Negative	Black		n/a	n/a	n/a
34	No_ATL_23	Training-No_ATL	<0.770	0.710	0	n/a	5.8	Missing data	HAM	No Rx	m	78.0	Unknown	Black		n/a	n/a	n/a
10	No_ATL_24	Training-No_ATL	<0.770	0.637	0	n/a	11.4	2.2	HAM	Methotrexate	f	60.0	Negative	Black		n/a	n/a	n/a
244	No_ATL_25	Training-No_ATL	<0.770	0.706	0	n/a	9.1	2.7	HAM	No Rx	f	74.0	Positive	Black		n/a	n/a	n/a
245	No_ATL_26	Training-No_ATL	<0.770	0.648	0	n/a	48.6	2.0	HAM	No Rx	f	65.0	Negative	Black		n/a	n/a	n/a
27	No_ATL_27	Training-No_ATL	<0.770	0.721	0	n/a	27.4	1.4	HAM	No Rx	m	71.0	Unknown	White		n/a	n/a	n/a
188	No_ATL_28	Training-No_ATL	<0.770	0.699	0	n/a	25.3	4.4****	HAM	Ciclosporin	f	52.0	Negative	White		n/a	n/a	n/a
14	No_ATL_29	Training-No_ATL	<0.770	0.708	0	n/a	34.7	5.2*****	HAM	No Rx	f	21.0	Negative	Black		n/a	n/a	n/a
15	No_ATL_30	Training-No_ATL	<0.770	0.628	0	n/a	6.7	1.6	HAM	No Rx	f	52.0	Negative	Black		n/a	n/a	n/a
33	No_ATL_31	Training-No_ATL	<0.770	0.714	0	n/a	9.4	2.0	HAM	Ciclosporin	f	50.0	Positive	Black		n/a	n/a	n/a
16	No_ATL_32	Training-No_ATL	<0.770	0.690	0	n/a	7.8	1.6	HAM	No Rx	f	60.0	Negative	Black		n/a	n/a	n/a
11	No_ATL_33	Training-No_ATL	<0.770	0.651	0	n/a	5.2	2.2	HAM	No Rx	f	52.0	Positive	Black		n/a	n/a	n/a
17	No_ATL_34	Training-No_ATL	<0.770	0.684	0	n/a	14.0	Missing data	HAM	No Rx	m	66.0	Positive	Black		n/a	n/a	n/a
18	No_ATL_35	Training-No_ATL	<0.770	0.623	0	n/a	4.0	2.7	HAM	No Rx	m	50.0	Positive	Black		n/a	n/a	n/a
12	No_ATL_36	Training-No_ATL	<0.770	0.692	0	n/a	38.6	3.3	HAM	No Rx	f	50.0	Negative	Black		n/a	n/a	n/a
29	No_ATL_37	Training-No_ATL	<0.770	0.726	0	n/a	3.7	1.5	HAM	No Rx	m	64.0	Negative	White		n/a	n/a	n/a
253	No_ATL_38	Training-No_ATL	<0.770	0.678	0	n/a	6.1	Missing data	HAM	No Rx	f	35.0	Negative	Black		n/a	n/a	n/a
269	Screening_1	Screening	<0.770	0.647	0	n/a	0.9	2.0	AC, HIV Co-infected	No Rx	f	55.0	Negative	Black		Did_not_transform	1.17	1.17
270	Screening_2	Screening	<0.770	0.660	0	n/a	0.2	2.3	AC, HIV Co-infected	No Rx	f	65.0	Unknown	Black		Did_not_transform	1.05	1.05
201	Screening_3	Screening	<0.770	0.656	0	n/a	22.0	2.6	AC, HIV Co-infected	No Rx	m	58.0	Unknown	Black		Did_not_transform	1.71	1.71
271	Screening_4	Screening	<0.770	0.614	0	n/a	1.2	Missing data	AC, HIV Co-infected	No Rx	m	60.0	Negative	Black		Did_not_transform	0.62	0.62
22	Screening_5	Screening	>0.770	0.886	2	21.0	14.3	Missing data	AC, HIV Co-infected	No Rx	f	59.0	Unknown	Black		Lost_to_followup	0.00	0.50
209	Screening_6	Screening	>0.770	0.797	1	5.2	13.7	2.5	AC, HIV Co-infected	No Rx	f	46.0	Unknown	Black		Did_not_transform	2.26	2.26
217	Screening_7	Screening	<0.770	0.625	0	n/a	4.2	Missing data	AC, HIV Co-infected	No Rx	m	73.0	Negative	White		Did_not_transform	1.27	1.27
203	Screening_8	Screening	<0.770	0.716	0	n/a	16.7	2.7	HAM, HIV Co-infected	Hydroxychloroquine	f	43.0	Unknown	Black		Did_not_transform	1.36	1.36
194	Screening_9	Screening	<0.770	0.686	0	n/a	14.5	3.0	AC, HIV Co-infected	No Rx	m	53.0	Unknown	Black		Lost_to_followup	0.00	0.50
204	Screening_10	Screening	<0.770	0.670	0	n/a	16.7	2.9	AC, HIV Co-infected	No Rx	f	40.0	Unknown	Black		Did_not_transform	1.07	1.07
99	Screening_11	Screening	<0.770	0.707	0	n/a	23.3	1.8	Arthritis	Etanercept	m	47.0	Unknown	White		Did_not_transform	1.69	1.69
89	Screening_12	Screening	<0.770	0.686	0	n/a	4.1	1.9	Arthritis	Methotrexate	f	55.0	Negative	Black		Did_not_transform	1.59	1.59
38	Screening_13	Screening	<0.770	0.728	0	n/a	5.6	1.6	AC	No Rx	f	75.0	Negative	Black	y	Lost_to_followup	4.00	4.50
116	Screening_14	Screening	<0.770	0.606	0	n/a	1.5	Missing data	AC	No Rx	f	25.0	Unknown	Black		Did_not_transform	1.07	1.07
178	Screening_15	Screening	<0.770	0.586	0	n/a	1.2	Missing data	AC	No Rx	f	35.0	Unknown	Black		Lost_to_followup	1.61	2.11
4	Screening_16	Screening	<0.770	0.674	0	n/a	0.1	4.6	AC	No Rx	f	40.0	Unknown	Black		Lost_to_followup	5.96	6.46
210	Screening_17	Screening	<0.770	0.749	0	n/a	12.9	Missing data	AC	No Rx	f	47.0	Unknown	Black		Lost_to_followup	0.00	0.50
218	Screening_18	Screening	<0.770	0.727	0	n/a	1.2	Missing data	AC	No Rx	f	68.0	Negative	Black		Lost_to_followup	3.58	4.08
90	Screening_19	Screening	<0.770	0.670	0	n/a	0.7	Missing data	AC	No Rx	f	53.0	Unknown	White		Did_not_transform	1.17	1.17
132	Screening_20	Screening	<0.770	0.672	0	n/a	2.9	1.0	AC	No Rx	f	60.0	Negative	Black		Did_not_transform	1.95	1.95
158	Screening_21	Screening	<0.770	0.687	0	n/a	0.1	1.9	AC	No Rx	m	67.0	Positive	Black		Lost_to_followup	0.00	0.50
91	Screening_22	Screening	<0.770	0.656	0	n/a	4.0	1.3	AC	No Rx	m	32.0	Negative	Black	possible	Did_not_transform	2.02	2.02
255	Screening_23	Screening	<0.770	0.762	0	n/a	16.6	3.8	AC	No Rx	f	29.0	Negative	Black		Did_not_transform	3.16	3.16
219	Screening_24	Screening	<0.770	0.697	0	n/a	1.2	Missing data	AC	No Rx	f	39.0	Unknown	White		Lost_to_followup	0.00	0.50
165	Screening_25	Screening	<0.770	0.607	0	n/a	4.5	Missing data	AC	No Rx	f	18.0	Positive	Black		Did_not_transform	11.32	11.32
148	Screening_26	Screening	<0.770	0.667	0	n/a	11.5	1.5	AC	No Rx	f	23.0	Negative	Black	possible	Lost_to_followup	0.88	1.38
103	Screening_27	Screening	<0.770	0.743	0	n/a	12.8	Missing data	AC	No Rx	f	65.0	Negative	Black		Did_not_transform	1.86	1.86
213	Screening_28	Screening	<0.770	0.673	0	n/a	11.6	2.6	AC	No Rx	f	61.0	Negative			Did_not_transform	2.27	2.27
180	Screening_29	Screening	<0.770	0.746	0	n/a	3.8	1.6	AC	No Rx	f	24.0	Unknown	White		Lost_to_followup	1.07	1.57
220	Screening_30	Screening	>0.770	0.837	1	12.7	18.8	2.1	AC	No Rx	f	28.0	Negative	Middle Eastern		Did_not_transform	4.30	4.30
221	Screening_31	Screening	<0.770	0.748	0	n/a	24.0	1.8	AC	No Rx	f	60.0	Negative	Black	possible	Lost_to_followup	0.00	0.50
181	Screening_32	Screening	<0.770	0.759	0	n/a	17.6	2.2	AC	No Rx	f	55.0	Unknown	Black		Lost_to_followup	0.00	0.50
214	Screening_33	Screening	<0.770	0.678	0	n/a	31.5	1.4	AC	No Rx	m	47.0	Unknown	Black		Lost_to_followup	0.00	0.50
119	Screening_34	Screening	<0.770	0.632	0	n/a	8.6	Missing data	AC	No Rx	f	65.0	Unknown	White		Did_not_transform	1.32	1.32
168	Screening_35	Screening	<0.770	0.728	0	n/a	0.9	Missing data	AC	No Rx	m	48.0	Negative	Black		Did_not_transform	7.70	7.70
104	Screening_36	Screening	<0.770	0.648	0	n/a	3.6	2.5	AC	No Rx	f	54.0	Negative	White		Did_not_transform	1.84	1.84
149	Screening_37	Screening	<0.770	0.650	0	n/a	2.9	2.5	AC	No Rx	f	50.0	Unknown	Japanese		Did_not_transform	1.99	1.99
134	Screening_38	Screening	<0.770	0.636	0	n/a	3.8	2.0	AC	No Rx	f	76.0	Negative	Black		Lost_to_followup	2.14	2.64
150	Screening_39	Screening	>0.770	0.805	1	11.9	28.3	1.6	AC	Tacrolimus/mycophenylate	m	32.0	Unknown	Middle Eastern		Lost_to_followup	0.00	0.50
105	Screening_40	Screening	>0.770	0.783	1	13.6	14.7	2.5	AC	No Rx	m	38.0	Negative	Middle Eastern		Did_not_transform	2.51	2.51
263	Screening_41	Screening	<0.770	0.676	0	n/a	0.8	1.6	AC	No Rx	f	75.0	Unknown	White		Did_not_transform	1.99	1.99
264	Screening_42	Screening	<0.770	0.690	0	n/a	0.6	1.5	AC	No Rx	f	33.0	Unknown	Middle Eastern	possible	Did_not_transform	1.99	1.99
249	Screening_43	Screening	>0.770	0.785	0	n/a	6.5	2.1	AC	No Rx	f	34.0	Unknown	Black		Lost_to_followup	0.00	0.50
265	Screening_44	Screening	<0.770	0.670	0	n/a	0.6	2.3	AC	No Rx	f	38.0	Negative	Other		Did_not_transform	2.72	2.72
151	Screening_45	Screening	<0.770	0.746	0	n/a	5.6	1.5	AC	No Rx	f	53.0	Unknown	Black		Did_not_transform	3.38	3.38
185	Screening_46	Screening	<0.770	0.717	0	n/a	3.2	Missing data	AC	No Rx	f	41.0	Unknown	Black		Lost_to_followup	0.00	0.50
266	Screening_47	Screening	<0.770	0.655	0	n/a	0.3	1.6	AC	No Rx	f	64.0	Negative	Black	y	Did_not_transform	0.27	0.27

186	Screening_48	Screening	<0.770	0.739	0	n/a	4.9	2.5	AC	No Rx	f	39.0	Negative	Black		Did_not_transform	1.10	1.10
195	Screening_49	Screening	<0.770	0.684	0	n/a	12.9	1.8	AC	No Rx	f	39.0	Unknown	Black	y	Lost_to_followup	0.00	0.50
257	Screening_50	Screening	<0.770	0.634	0	n/a	2.8	Missing data	AC	No Rx	m	80.0	Unknown	Black		Did_not_transform	0.52	0.52
135	Screening_51	Screening	<0.770	0.652	0	n/a	2.3	2.5	AC	No Rx	f	52.0	Negative	Middle Eastern		Did_not_transform	1.00	1.00
273	Screening_52	Screening	<0.770	0.622	0	n/a	0.3	2.0	AC	No Rx	f	83.0	Negative	Black		Did_not_transform	1.57	1.57
92	Screening_53	Screening	<0.770	0.754	0	n/a	9.5	1.2	AC	No Rx	f	66.0	Negative	Black		Did_not_transform	1.63	1.63
274	Screening_54	Screening	<0.770	0.613	0	n/a	0.6	1.1	AC	No Rx	f	57.0	Unknown	Other		Did_not_transform	1.41	1.41
187	Screening_55	Screening	<0.770	0.730	0	n/a	5.2	2.4	AC	No Rx	f	53.0	Negative	Black		Did_not_transform	2.27	2.27
196	Screening_56	Screening	>0.770	0.834	1	18.0	18.4	Missing data	AC	No Rx	f	57.0	Unknown	Black	y	Did_not_transform	1.38	1.38
275	Screening_57	Screening	<0.770	0.598	0	n/a	0.6	Missing data	AC	No Rx	f	28.0	Unknown	Middle Eastern		Did_not_transform	0.55	0.55
56	Screening_58	Screening	>0.770	0.846	1	28.3	25.6	4.8****	AC	No Rx	f	48.0	Unknown	Black	y	Transformed	5.82	5.82
260	Screening_59	Screening	>0.770	0.985	1	62.8	20.5	4.4****	AC	No Rx	f	76.0	Unknown	Black	y	Transformed	3.45	3.45
70	Screening_60	Screening	>0.770	0.880	2	18.1	15.5	Missing data	AC	No Rx	m	51.0	Negative	Black	y	Treated	3.93	exclude
159	Screening_61	Screening	<0.770	0.586	0	n/a	4.0	1.4	Neurological	No Rx	f	53.0	Unknown	Black		Did_not_transform	2.77	2.77
152	Screening_62	Screening	<0.770	0.768	0	n/a	1.4	2.2	Neurological	No Rx	m	51.0	Negative	Black		Lost_to_followup	0.00	0.50
153	Screening_63	Screening	<0.770	0.769	0	n/a	9.1	Missing data	Neurological	No Rx	m	76.0	Unknown	Black		Lost_to_followup	0.17	0.67
154	Screening_64	Screening	<0.770	0.668	0	n/a	5.0	1.6	Neurological	No Rx	f	43.0	Negative	Black		Lost_to_followup	0.00	0.50
120	Screening_65	Screening	<0.770	0.689	0	n/a	11.6	1.5	Neurological	No Rx	f	63.0	Negative	Black		Did_not_transform	1.33	1.33
93	Screening_66	Screening	>0.770	0.795	1	3.0	8.3	2.6	Neurological	No Rx	f	63.0	Unknown	Black	y	Did_not_transform	1.35	1.35
160	Screening_67	Screening	>0.770	0.837	1	12.4	11.9	2.5	Strongyloides	No Rx	m	46.0	Positive	Black		Lost_to_followup	1.25	1.75
137	Screening_68	Screening	<0.770	0.716	0	n/a	27.0	2.0	Strongyloides	No Rx	f	56.0	Positive	Other		Did_not_transform	1.80	1.80
35	Screening_69	Screening	<0.770	0.706	0	n/a	6.0	Missing data	HAM	No Rx	f	53.0	Positive	Black		Lost_to_followup	2.55	3.05
155	Screening_70	Screening	<0.770	0.660	0	n/a	2.8	Missing data	HAM	No Rx	f	86.0	Negative	Black		Lost_to_followup	0.50	1.00
211	Screening_71	Screening	<0.770	0.701	0	n/a	17.7	Missing data	HAM	No Rx	m	73.0	Unknown	Indian/Pakistani/Bangladeshi		Lost_to_followup	0.00	0.50
30	Screening_72	Screening	<0.770	0.765	1	5.8	22.7	1.6	HAM	No Rx	f	73.0	Negative	Black		Lost_to_followup	4.01	4.51
189	Screening_73	Screening	<0.770	0.698	0	n/a	20.6	1.2	HAM	No Rx	f	78.0	Negative	Black		Lost_to_followup	0.28	0.78
45	Screening_74	Screening	<0.770	0.690	0	n/a	2.9	2.9	HAM	Hydroxychloroquine	m	73.0	Negative	White		Did_not_transform	1.79	1.79
118	Screening_75	Screening	<0.770	0.687	0	n/a	10.5	2.2	HAM	No Rx	f	70.0	Negative	Black		Did_not_transform	1.03	1.03
123	Screening_76	Screening	<0.770	0.662	0	n/a	8.9	1.4	HAM	Hydroxychloroquine	f	64.0	Negative	Black		Did_not_transform	1.27	1.27
190	Screening_77	Screening	<0.770	0.700	1	4.0	1.3	2.7	HAM	Ciclosporin	m	70.0	Negative	Black		Lost_to_followup	0.00	0.50
174	Screening_78	Screening	>0.770	0.782	0	n/a	21.8	3.4	HAM	Prednisolone/Hydroxychloroquine/Mycophenolate Mofetil	m	51.0	Negative	Black		Did_not_transform	1.59	1.59
110	Screening_79	Screening	<0.770	0.667	0	n/a	7.1	1.5	HAM	No Rx	f	59.0	Negative	Other		Did_not_transform	1.61	1.61
212	Screening_80	Screening	<0.770	0.694	0	n/a	22.0	3.2	HAM	No Rx	f	51.0	Negative	White		Did_not_transform	3.69	3.69
191	Screening_81	Screening	<0.770	0.695	0	n/a	4.9	1.8	HAM	No Rx	f	67.0	Negative	Black		Did_not_transform	1.90	1.90
126	Screening_82	Screening	<0.770	0.698	0	n/a	16.1	2.0	HAM	No Rx	f	46.0	Positive	Indian/Pakistani/Bangladeshi		Did_not_transform	1.30	1.30
111	Screening_83	Screening	<0.770	0.633	0	n/a	7.4	2.9	HAM	No Rx	f	63.0	Negative	Black		Did_not_transform	1.84	1.84
127	Screening_84	Screening	<0.770	0.590	0	n/a	13.0	3.4	HAM	No Rx	f	57.0	Negative	Other		Did_not_transform	1.88	1.88
156	Screening_85	Screening	<0.770	0.645	0	n/a	5.7	Missing data	HAM	Methotrexate	f	48.0	Positive	Black		Lost_to_followup	4.85	5.35
112	Screening_86	Screening	<0.770	0.679	0	n/a	17.1	1.6	HAM	No Rx	f	64.0	Unknown	Black		Did_not_transform	1.25	1.25
128	Screening_87	Screening	<0.770	0.633	0	n/a	10.3	2.3	HAM	Methotrexate	f	61.0	Negative	Black		Did_not_transform	0.92	0.92
129	Screening_88	Screening	<0.770	0.669	0	n/a	9.1	2.5	HAM	Methotrexate/Prednisolone	m	61.0	Negative	Black		Did_not_transform	1.86	1.86
141	Screening_89	Screening	<0.770	0.710	0	n/a	19.1	1.6	HAM	Methotrexate	m	66.0	Negative	Black		Did_not_transform	0.08	0.08
142	Screening_90	Screening	<0.770	0.617	0	n/a	8.9	2.6	HAM	No Rx	m	53.0	Unknown	Black		Did_not_transform	1.02	1.02
200	Screening_91	Screening	<0.770	0.699	0	n/a	24.2	1.3	HAM	Methotrexate	f	52.0	Negative	Black	possible	Did_not_transform	1.26	1.26
143	Screening_92	Screening	<0.770	0.729	0	n/a	6.6	1.9	HAM	No Rx	f	73.0	Negative	Black		Did_not_transform	1.25	1.25
76	Screening_93	Screening	<0.770	0.745	0	n/a	7.7	1.9	HAM	No Rx	f	50.0	Negative	Black		Did_not_transform	2.45	2.45
97	Screening_94	Screening	<0.770	0.623	0	n/a	10.8	1.8	HAM	Methotrexate	f	76.0	Negative	Black		Did_not_transform	1.58	1.58
197	Screening_95	Screening	<0.770	0.749	1	8.7	9.7	Missing data	HAM	No Rx	f	63.0	Positive	Black		Lost_to_followup	0.00	0.50
157	Screening_96	Screening	<0.770	0.722	0	n/a	20.5	3.1	HAM	No Rx	m	54.0	Negative	Black		Did_not_transform	4.00	4.00
144	Screening_97	Screening	<0.770	0.682	0	n/a	8.5	1.7	HAM	Methotrexate	m	53.0	Unknown	Black		Did_not_transform	1.70	1.70
113	Screening_98	Screening	>0.770	0.788	2	15.7	31.5	3.2	HAM	No Rx	f	55.0	Negative	Black		Did_not_transform	1.65	1.65
192	Screening_99	Screening	<0.770	0.647	0	n/a	15.3	1.8	HAM	No Rx	m	81.0	Negative	Black		Lost_to_followup	0.00	0.50
130	Screening_100	Screening	<0.770	0.715	0	n/a	14.2	1.3	HAM	Methotrexate	m	63.0	Unknown	Black		Did_not_transform	1.21	1.21
145	Screening_101	Screening	<0.770	0.688	0	n/a	8.6	3.0	HAM	No Rx	f	52.0	Negative	Black		Did_not_transform	1.39	1.39
268	Screening_102	Screening	<0.770	0.695	0	n/a	1.2	0.8	HAM	Methotrexate/Prednisolone	m	79.0	Negative	White		Did_not_transform	1.36	1.36
114	Screening_103	Screening	<0.770	0.746	0	n/a	37.4	2.5	HAM	No Rx	f	68.0	Negative	Black		Did_not_transform	2.26	2.26
276	Screening_104	Screening	<0.770	0.662	0	n/a	1.9	Missing data	HAM	No Rx	m	61.0	Unknown	Black		Did_not_transform	0.52	0.52
146	Screening_105	Screening	<0.770	0.709	0	n/a	12.4	2.3	HAM	No Rx	f	64.0	Unknown	Middle Eastern		Did_not_transform	1.28	1.28
193	Screening_106	Screening	<0.770	0.695	0	n/a	0.6	Missing data	Uveitis	No Rx	f	89.0	Unknown	Black		Lost_to_followup	0.00	0.50

* abbreviations

** Lymphocyte count 5 months pre OCI-flow assay; 6 months pre treatment

*** Lymphocyte count from 2 months pre OCI-flow assay

**** Transient lymphocytosis

***** Occasional lymphocyte count >4x10^9/L, not persistent
