# Supplementary material for: Quantification of T cell clonality in human T cell leukaemia virus type-1 carriers can detect the development of adult T cell leukaemia early
Source: Blood Cancer J. 2021 Mar 26;11(3):66. doi: 10.1038/s41408-021-00458-8 (PMC7997885; doi:10.1038/s41408-021-00458-8)
Supplement: Supplementary file 2 — Supplemental material [file 41408_2021_458_MOESM2_ESM.pptx]

## Slide 1
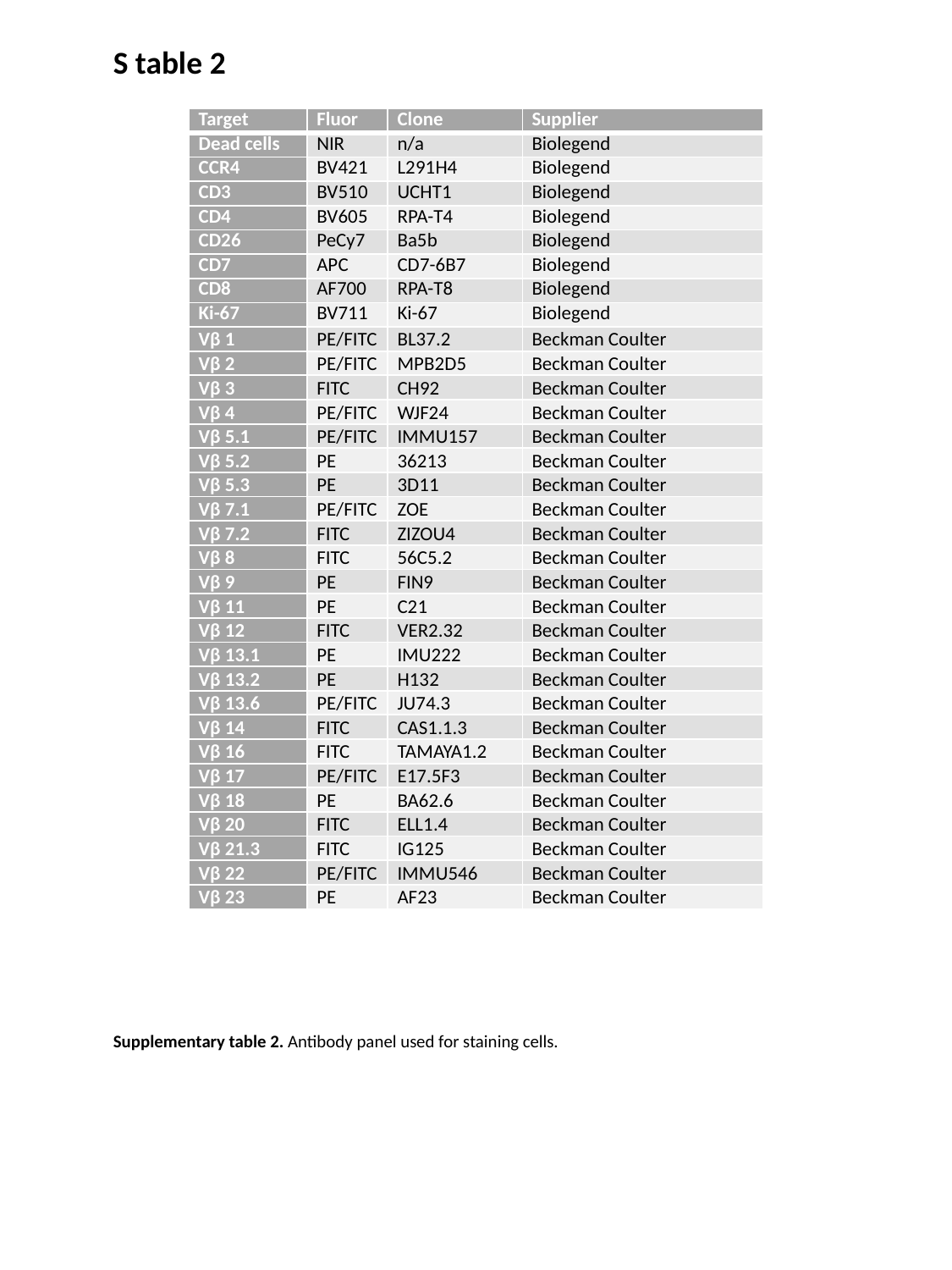

S table 2
| Target | Fluor | Clone | Supplier |
| --- | --- | --- | --- |
| Dead cells | NIR | n/a | Biolegend |
| CCR4 | BV421 | L291H4 | Biolegend |
| CD3 | BV510 | UCHT1 | Biolegend |
| CD4 | BV605 | RPA-T4 | Biolegend |
| CD26 | PeCy7 | Ba5b | Biolegend |
| CD7 | APC | CD7-6B7 | Biolegend |
| CD8 | AF700 | RPA-T8 | Biolegend |
| Ki-67 | BV711 | Ki-67 | Biolegend |
| Vβ 1 | PE/FITC | BL37.2 | Beckman Coulter |
| Vβ 2 | PE/FITC | MPB2D5 | Beckman Coulter |
| Vβ 3 | FITC | CH92 | Beckman Coulter |
| Vβ 4 | PE/FITC | WJF24 | Beckman Coulter |
| Vβ 5.1 | PE/FITC | IMMU157 | Beckman Coulter |
| Vβ 5.2 | PE | 36213 | Beckman Coulter |
| Vβ 5.3 | PE | 3D11 | Beckman Coulter |
| Vβ 7.1 | PE/FITC | ZOE | Beckman Coulter |
| Vβ 7.2 | FITC | ZIZOU4 | Beckman Coulter |
| Vβ 8 | FITC | 56C5.2 | Beckman Coulter |
| Vβ 9 | PE | FIN9 | Beckman Coulter |
| Vβ 11 | PE | C21 | Beckman Coulter |
| Vβ 12 | FITC | VER2.32 | Beckman Coulter |
| Vβ 13.1 | PE | IMU222 | Beckman Coulter |
| Vβ 13.2 | PE | H132 | Beckman Coulter |
| Vβ 13.6 | PE/FITC | JU74.3 | Beckman Coulter |
| Vβ 14 | FITC | CAS1.1.3 | Beckman Coulter |
| Vβ 16 | FITC | TAMAYA1.2 | Beckman Coulter |
| Vβ 17 | PE/FITC | E17.5F3 | Beckman Coulter |
| Vβ 18 | PE | BA62.6 | Beckman Coulter |
| Vβ 20 | FITC | ELL1.4 | Beckman Coulter |
| Vβ 21.3 | FITC | IG125 | Beckman Coulter |
| Vβ 22 | PE/FITC | IMMU546 | Beckman Coulter |
| Vβ 23 | PE | AF23 | Beckman Coulter |
Supplementary table 2. Antibody panel used for staining cells.

## Slide 2
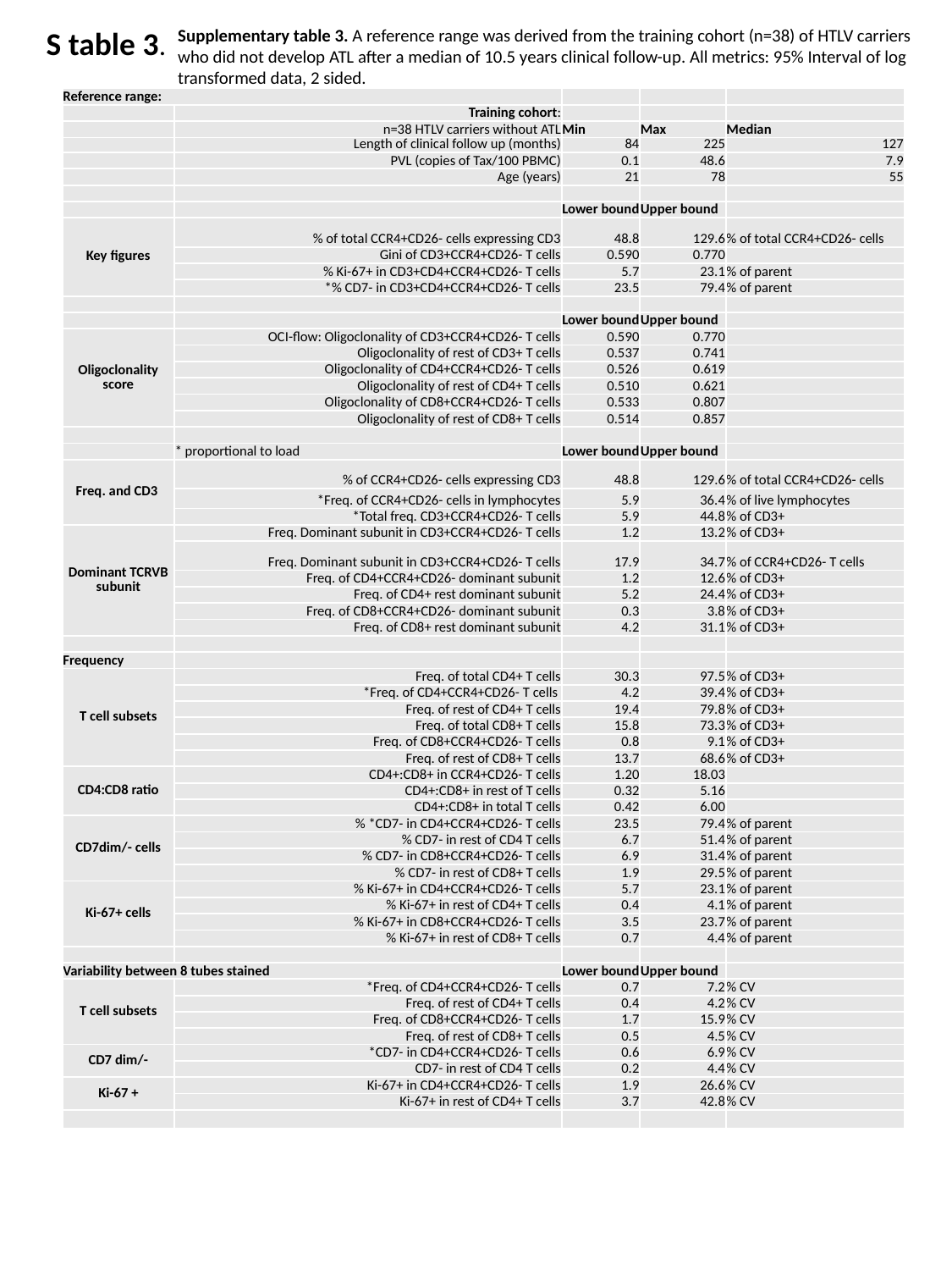

S table 3.
Supplementary table 3. A reference range was derived from the training cohort (n=38) of HTLV carriers who did not develop ATL after a median of 10.5 years clinical follow-up. All metrics: 95% Interval of log transformed data, 2 sided.
| Reference range: | | | | |
| --- | --- | --- | --- | --- |
| | Training cohort: | | | |
| | n=38 HTLV carriers without ATL | Min | Max | Median |
| | Length of clinical follow up (months) | 84 | 225 | 127 |
| | PVL (copies of Tax/100 PBMC) | 0.1 | 48.6 | 7.9 |
| | Age (years) | 21 | 78 | 55 |
| | | | | |
| | | Lower bound | Upper bound | |
| Key figures | % of total CCR4+CD26- cells expressing CD3 | 48.8 | 129.6 | % of total CCR4+CD26- cells |
| | Gini of CD3+CCR4+CD26- T cells | 0.590 | 0.770 | |
| | % Ki-67+ in CD3+CD4+CCR4+CD26- T cells | 5.7 | 23.1 | % of parent |
| | \*% CD7- in CD3+CD4+CCR4+CD26- T cells | 23.5 | 79.4 | % of parent |
| | | | | |
| | | Lower bound | Upper bound | |
| Oligoclonality score | OCI-flow: Oligoclonality of CD3+CCR4+CD26- T cells | 0.590 | 0.770 | |
| | Oligoclonality of rest of CD3+ T cells | 0.537 | 0.741 | |
| | Oligoclonality of CD4+CCR4+CD26- T cells | 0.526 | 0.619 | |
| | Oligoclonality of rest of CD4+ T cells | 0.510 | 0.621 | |
| | Oligoclonality of CD8+CCR4+CD26- T cells | 0.533 | 0.807 | |
| | Oligoclonality of rest of CD8+ T cells | 0.514 | 0.857 | |
| | | | | |
| | \* proportional to load | Lower bound | Upper bound | |
| Freq. and CD3 | % of CCR4+CD26- cells expressing CD3 | 48.8 | 129.6 | % of total CCR4+CD26- cells |
| | \*Freq. of CCR4+CD26- cells in lymphocytes | 5.9 | 36.4 | % of live lymphocytes |
| | \*Total freq. CD3+CCR4+CD26- T cells | 5.9 | 44.8 | % of CD3+ |
| Dominant TCRVB subunit | Freq. Dominant subunit in CD3+CCR4+CD26- T cells | 1.2 | 13.2 | % of CD3+ |
| | Freq. Dominant subunit in CD3+CCR4+CD26- T cells | 17.9 | 34.7 | % of CCR4+CD26- T cells |
| | Freq. of CD4+CCR4+CD26- dominant subunit | 1.2 | 12.6 | % of CD3+ |
| | Freq. of CD4+ rest dominant subunit | 5.2 | 24.4 | % of CD3+ |
| | Freq. of CD8+CCR4+CD26- dominant subunit | 0.3 | 3.8 | % of CD3+ |
| | Freq. of CD8+ rest dominant subunit | 4.2 | 31.1 | % of CD3+ |
| | | | | |
| Frequency | | | | |
| T cell subsets | Freq. of total CD4+ T cells | 30.3 | 97.5 | % of CD3+ |
| | \*Freq. of CD4+CCR4+CD26- T cells | 4.2 | 39.4 | % of CD3+ |
| | Freq. of rest of CD4+ T cells | 19.4 | 79.8 | % of CD3+ |
| | Freq. of total CD8+ T cells | 15.8 | 73.3 | % of CD3+ |
| | Freq. of CD8+CCR4+CD26- T cells | 0.8 | 9.1 | % of CD3+ |
| | Freq. of rest of CD8+ T cells | 13.7 | 68.6 | % of CD3+ |
| CD4:CD8 ratio | CD4+:CD8+ in CCR4+CD26- T cells | 1.20 | 18.03 | |
| | CD4+:CD8+ in rest of T cells | 0.32 | 5.16 | |
| | CD4+:CD8+ in total T cells | 0.42 | 6.00 | |
| CD7dim/- cells | % \*CD7- in CD4+CCR4+CD26- T cells | 23.5 | 79.4 | % of parent |
| | % CD7- in rest of CD4 T cells | 6.7 | 51.4 | % of parent |
| | % CD7- in CD8+CCR4+CD26- T cells | 6.9 | 31.4 | % of parent |
| | % CD7- in rest of CD8+ T cells | 1.9 | 29.5 | % of parent |
| Ki-67+ cells | % Ki-67+ in CD4+CCR4+CD26- T cells | 5.7 | 23.1 | % of parent |
| | % Ki-67+ in rest of CD4+ T cells | 0.4 | 4.1 | % of parent |
| | % Ki-67+ in CD8+CCR4+CD26- T cells | 3.5 | 23.7 | % of parent |
| | % Ki-67+ in rest of CD8+ T cells | 0.7 | 4.4 | % of parent |
| | | | | |
| Variability between 8 tubes stained | | Lower bound | Upper bound | |
| T cell subsets | \*Freq. of CD4+CCR4+CD26- T cells | 0.7 | 7.2 | % CV |
| | Freq. of rest of CD4+ T cells | 0.4 | 4.2 | % CV |
| | Freq. of CD8+CCR4+CD26- T cells | 1.7 | 15.9 | % CV |
| | Freq. of rest of CD8+ T cells | 0.5 | 4.5 | % CV |
| CD7 dim/- | \*CD7- in CD4+CCR4+CD26- T cells | 0.6 | 6.9 | % CV |
| | CD7- in rest of CD4 T cells | 0.2 | 4.4 | % CV |
| Ki-67 + | Ki-67+ in CD4+CCR4+CD26- T cells | 1.9 | 26.6 | % CV |
| | Ki-67+ in rest of CD4+ T cells | 3.7 | 42.8 | % CV |
| | | | | |

## Slide 3
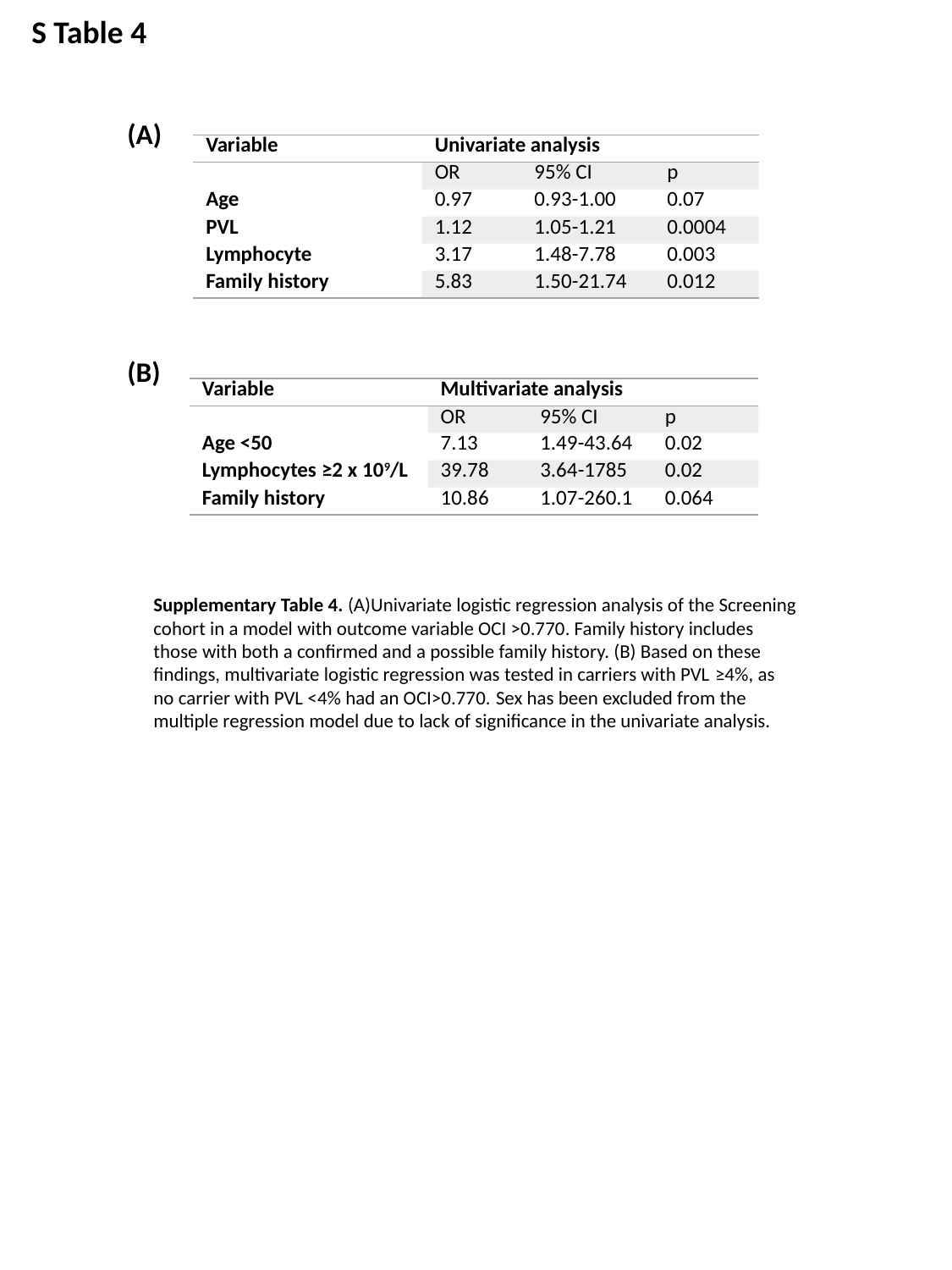

S Table 4
(A)
| Variable | Univariate analysis | | |
| --- | --- | --- | --- |
| | OR | 95% CI | p |
| Age | 0.97 | 0.93-1.00 | 0.07 |
| PVL | 1.12 | 1.05-1.21 | 0.0004 |
| Lymphocyte | 3.17 | 1.48-7.78 | 0.003 |
| Family history | 5.83 | 1.50-21.74 | 0.012 |
(B)
| Variable | Multivariate analysis | | |
| --- | --- | --- | --- |
| | OR | 95% CI | p |
| Age <50 | 7.13 | 1.49-43.64 | 0.02 |
| Lymphocytes ≥2 x 10^9^/L | 39.78 | 3.64-1785 | 0.02 |
| Family history | 10.86 | 1.07-260.1 | 0.064 |
Supplementary Table 4. (A)Univariate logistic regression analysis of the Screening cohort in a model with outcome variable OCI >0.770. Family history includes those with both a confirmed and a possible family history. (B) Based on these findings, multivariate logistic regression was tested in carriers with PVL ≥4%, as no carrier with PVL <4% had an OCI>0.770. Sex has been excluded from the multiple regression model due to lack of significance in the univariate analysis.

## Slide 4
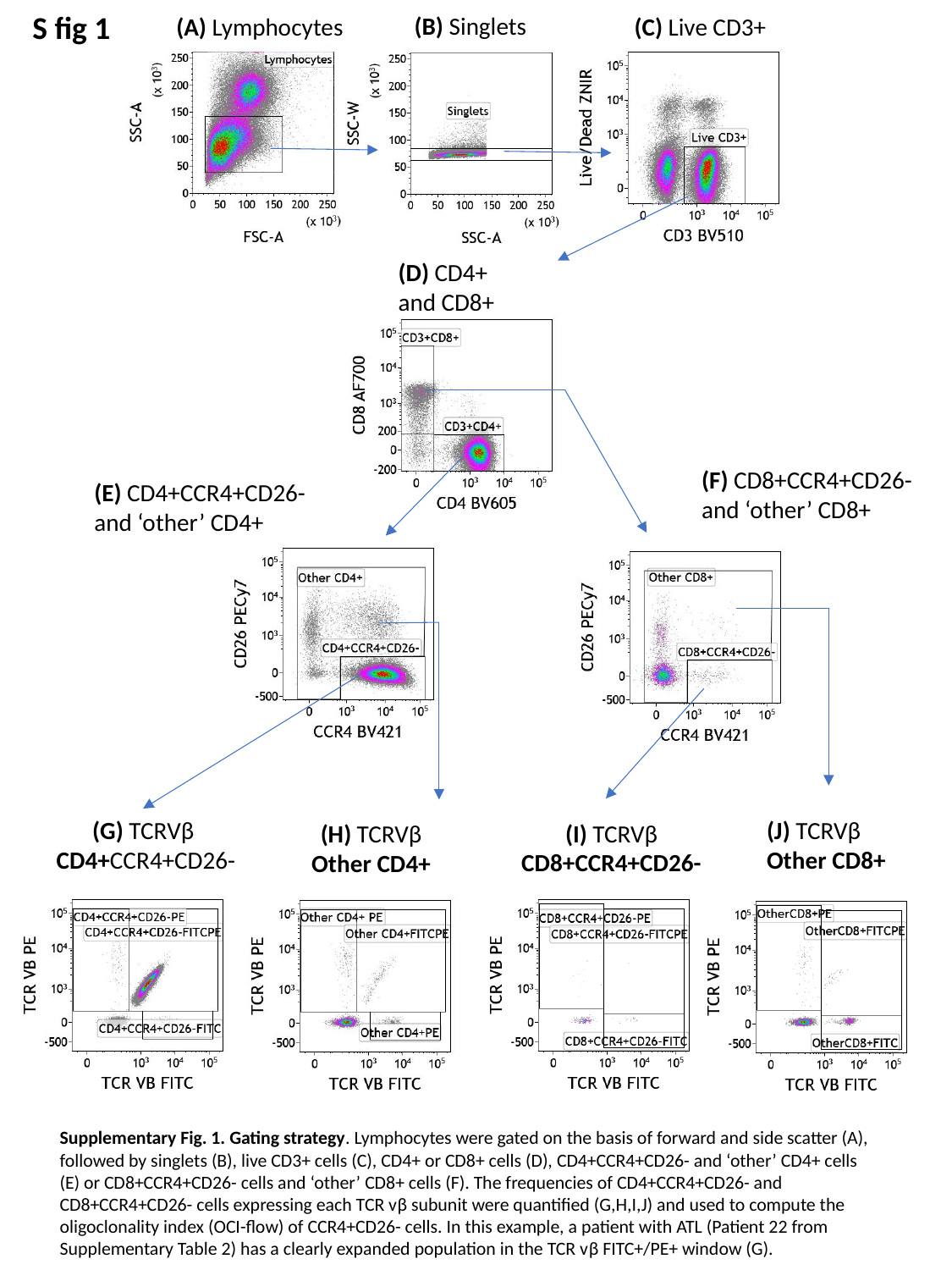

S fig 1
(B) Singlets
(C) Live CD3+
(A) Lymphocytes
(D) CD4+
and CD8+
(F) CD8+CCR4+CD26-
and ‘other’ CD8+
(E) CD4+CCR4+CD26-
and ‘other’ CD4+
(J) TCRVβ
Other CD8+
(G) TCRVβ
 CD4+CCR4+CD26-
(I) TCRVβ
CD8+CCR4+CD26-
(H) TCRVβ
Other CD4+
Supplementary Fig. 1. Gating strategy. Lymphocytes were gated on the basis of forward and side scatter (A), followed by singlets (B), live CD3+ cells (C), CD4+ or CD8+ cells (D), CD4+CCR4+CD26- and ‘other’ CD4+ cells (E) or CD8+CCR4+CD26- cells and ‘other’ CD8+ cells (F). The frequencies of CD4+CCR4+CD26- and CD8+CCR4+CD26- cells expressing each TCR vβ subunit were quantified (G,H,I,J) and used to compute the oligoclonality index (OCI-flow) of CCR4+CD26- cells. In this example, a patient with ATL (Patient 22 from Supplementary Table 2) has a clearly expanded population in the TCR vβ FITC+/PE+ window (G).

## Slide 5
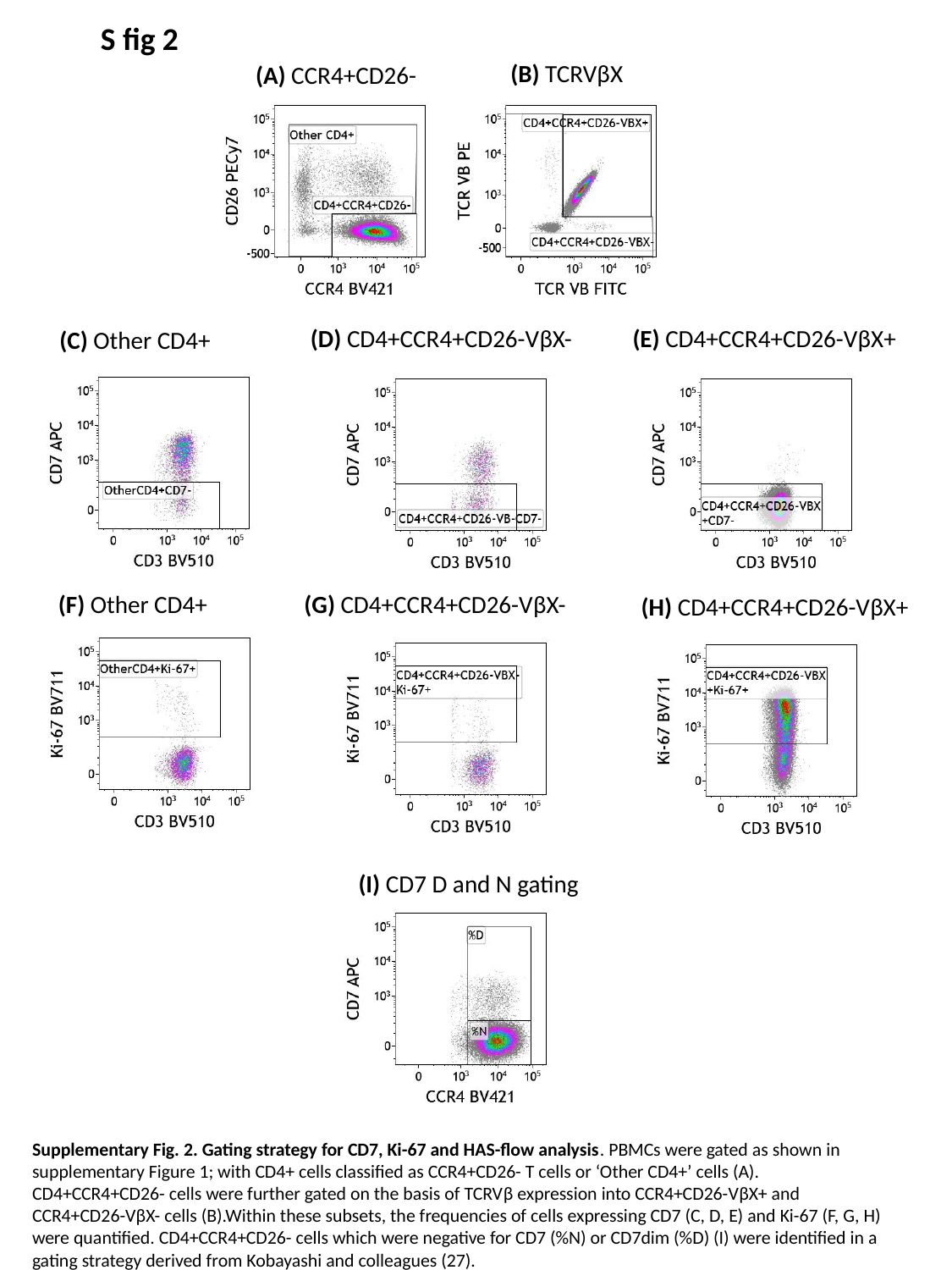

# S fig 2
(B) TCRVβX
(A) CCR4+CD26-
(C) Other CD4+
| (E) CD4+CCR4+CD26-VβX+ |
| --- |
| (D) CD4+CCR4+CD26-VβX- |
| --- |
(F) Other CD4+
| (G) CD4+CCR4+CD26-VβX- |
| --- |
| (H) CD4+CCR4+CD26-VβX+ |
| --- |
(I) CD7 D and N gating
Supplementary Fig. 2. Gating strategy for CD7, Ki-67 and HAS-flow analysis. PBMCs were gated as shown in supplementary Figure 1; with CD4+ cells classified as CCR4+CD26- T cells or ‘Other CD4+’ cells (A). CD4+CCR4+CD26- cells were further gated on the basis of TCRVβ expression into CCR4+CD26-VβX+ and CCR4+CD26-VβX- cells (B).Within these subsets, the frequencies of cells expressing CD7 (C, D, E) and Ki-67 (F, G, H) were quantified. CD4+CCR4+CD26- cells which were negative for CD7 (%N) or CD7dim (%D) (I) were identified in a gating strategy derived from Kobayashi and colleagues (27).

## Slide 6
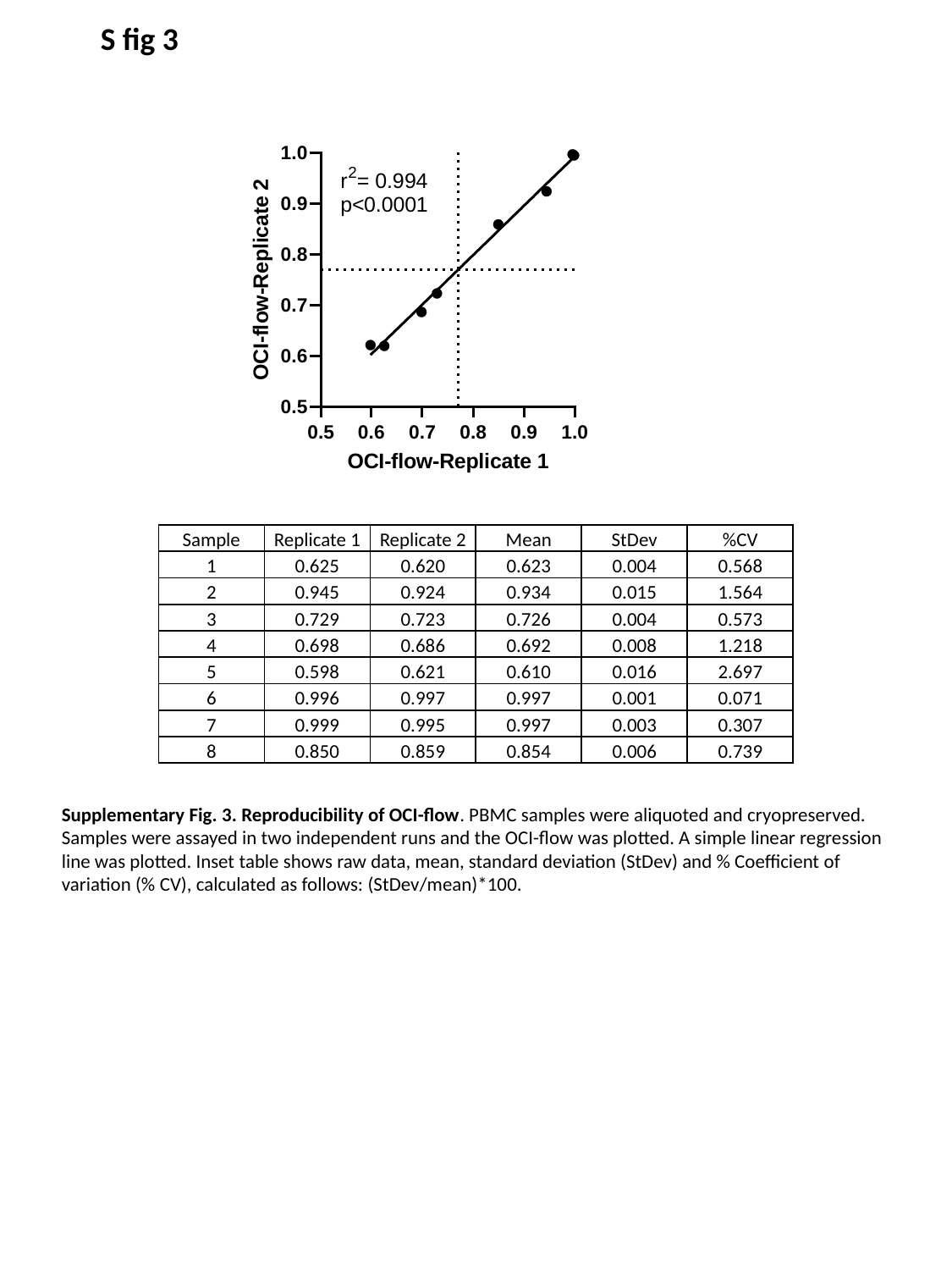

S fig 3
| Sample | Replicate 1 | Replicate 2 | Mean | StDev | %CV |
| --- | --- | --- | --- | --- | --- |
| 1 | 0.625 | 0.620 | 0.623 | 0.004 | 0.568 |
| 2 | 0.945 | 0.924 | 0.934 | 0.015 | 1.564 |
| 3 | 0.729 | 0.723 | 0.726 | 0.004 | 0.573 |
| 4 | 0.698 | 0.686 | 0.692 | 0.008 | 1.218 |
| 5 | 0.598 | 0.621 | 0.610 | 0.016 | 2.697 |
| 6 | 0.996 | 0.997 | 0.997 | 0.001 | 0.071 |
| 7 | 0.999 | 0.995 | 0.997 | 0.003 | 0.307 |
| 8 | 0.850 | 0.859 | 0.854 | 0.006 | 0.739 |
Supplementary Fig. 3. Reproducibility of OCI-flow. PBMC samples were aliquoted and cryopreserved. Samples were assayed in two independent runs and the OCI-flow was plotted. A simple linear regression line was plotted. Inset table shows raw data, mean, standard deviation (StDev) and % Coefficient of variation (% CV), calculated as follows: (StDev/mean)*100.

## Slide 7
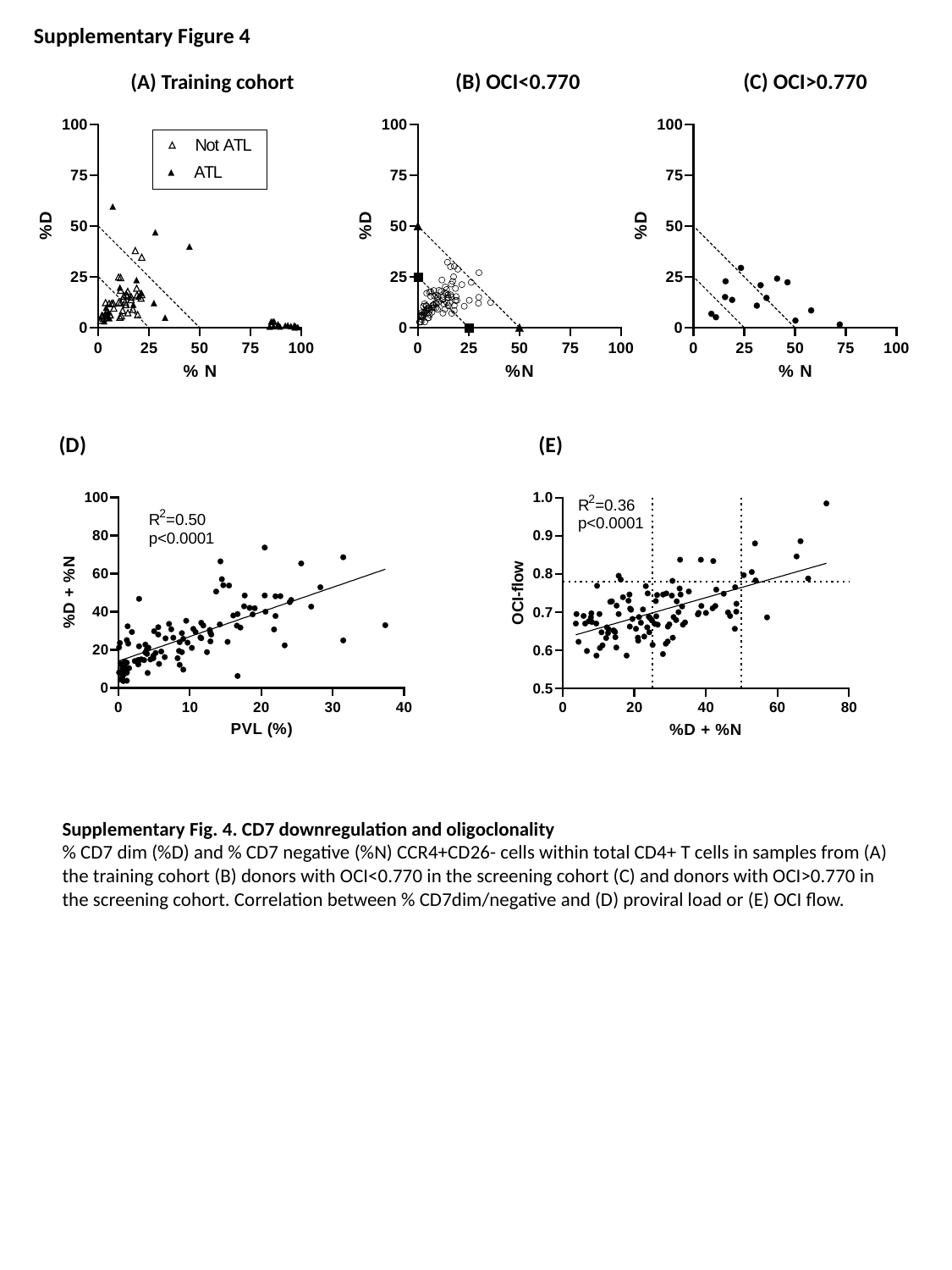

Supplementary Figure 4
(B) OCI<0.770
(C) OCI>0.770
(A) Training cohort
(D)
(E)
Supplementary Fig. 4. CD7 downregulation and oligoclonality
% CD7 dim (%D) and % CD7 negative (%N) CCR4+CD26- cells within total CD4+ T cells in samples from (A) the training cohort (B) donors with OCI<0.770 in the screening cohort (C) and donors with OCI>0.770 in the screening cohort. Correlation between % CD7dim/negative and (D) proviral load or (E) OCI flow.
